# Rapid intestinal and systemic metabolic reprogramming in an immunosuppressed environment

**DOI:** 10.1186/s12866-023-03141-z

**Published:** 2023-12-09

**Authors:** Bing Ma, Samuel J. Gavzy, Michael France, Yang Song, Hnin Wai Lwin, Allison Kensiski, Vikas Saxena, Wenji Piao, Ram Lakhan, Jegan Iyyathurai, Lushen Li, Christina Paluskievicz, Long Wu, Marina WillsonShirkey, Emmanuel F. Mongodin, Valeria R. Mas, Jonathan S. Bromberg

**Affiliations:** 1 Institute of Genome Sciences, University of Maryland School of Medicine, Baltimore, MD 21201 USA; 2 Department of Microbiology and Immunology, University of Maryland School of Medicine, Baltimore, MD 21201 USA; 3 Department of Surgery, University of Maryland Medical Center, Baltimore, MD 21201 USA; 4 Center for Vascular and Inflammatory Diseases, University of Maryland School of Medicine, Baltimore, MD 21201 USA; 5 Present Address: Division of Lung Diseases, National Heart, Lung, and Blood Institute (NHLBI), National Institutes of Health (NIH), Bethesda, MD USA

**Keywords:** Gut microbiome, Metabolome, Immune tolerance, Immunosuppression, Transplantation, Antibiotics, Gut dysbiosis, Integrative multi-omics

## Abstract

Intrinsic metabolism shapes the immune environment associated with immune suppression and tolerance in settings such as organ transplantation and cancer. However, little is known about the metabolic activities in an immunosuppressive environment. In this study, we employed metagenomic, metabolomic, and immunological approaches to profile the early effects of the immunosuppressant drug tacrolimus, antibiotics, or both in gut lumen and circulation using a murine model. Tacrolimus induced rapid and profound alterations in metabolic activities within two days of treatment, prior to alterations in gut microbiota composition and structure. The metabolic profile and gut microbiome after seven days of treatment was distinct from that after two days of treatment, indicating continuous drug effects on both gut microbial ecosystem and host metabolism. The most affected taxonomic groups are Clostriales and Verrucomicrobiae (i.e., *Akkermansia muciniphila*), and the most affected metabolic pathways included a group of interconnected amino acids, bile acid conjugation, glucose homeostasis, and energy production. Highly correlated metabolic changes were observed between lumen and serum metabolism, supporting their significant interactions. Despite a small sample size, this study explored the largely uncharacterized microbial and metabolic events in an immunosuppressed environment and demonstrated that early changes in metabolic activities can have significant implications that may serve as antecedent biomarkers of immune activation or quiescence. To understand the intricate relationships among gut microbiome, metabolic activities, and immune cells in an immune suppressed environment is a prerequisite for developing strategies to monitor and optimize alloimmune responses that determine transplant outcomes.

**Supplementary Information:**

The online version contains supplementary material available at 10.1186/s12866-023-03141-z.

## Introduction

The intrinsic metabolism is a major regulator of the immune environment, including metabolic activities associated with immune tolerance such as in transplantation and cancer [1–3]. The immunosuppressive agents used to prevent allograft rejection have serious long-term effects not only on immunity and the transplanted organ, but also on metabolic disorders [4]. Metabolic derangements such as post-transplant diabetes mellitus, non-alcoholic fatty liver disease, hypertension, dyslipidemia, and obesity affect the majority of organ transplant recipients [5]. As major non-immune factors, metabolic disorders contribute significantly to chronic allograft dysfunction, graft survival, and quality of life [6–8]. Gut dysbiosis appears also to be involved in these metabolic changes, though its specific impact remains undefined [9]. Despite of current understanding of a few metabolites implicated in cancer immunosuppression [9, 10], there is a significant gap in our understanding of the shifts in the metabolic landscape and the specific microbiomes that are responsible for such changes. Notably, many metabolic changes have been characterized after the emergence of symptomatic pathophysiological signs, leaving the early shifts less explored.

Many metabolic disorders are characterized as an imbalanced composition and function of intestinal microbiota with reduced microbial biodiversity and altered metabolic capacity, or gut dysbiosis [11, 12]. In a dysbiotic state, microbial metabolic activities are altered and a large range of metabolites are affected, such as amino acids [13], short-chain fatty acids (SCFAs) [14], bile acids (BAs) [15], tryptophan metabolites [16], trimethylamine N-oxide (TMAO) [17], polyamines [18], and vitamin derivatives [19]. Dysbiosis can result in an overgrowth of potentially harmful microbes that produce proinflammatory metabolites, such as lipopolysaccharides and TMAO [20]. Increased levels of these metabolites contribute to systemic inflammation, impaired gut barrier function, and immune dysregulation [21]. In fact, gut dysbiosis and altered metabolic pathways are associated with increased mortality after organ transplantation [12]. Ameliorating gut dysbiosis using dietary interventions, prebiotics, probiotics, and fecal microbiota transplantation offers potential prevention and treatment of metabolic disorders.

Interactions between immunosuppressive treatments and gut microbiome are bidirectional. Tacrolimus is a widely used immunosuppressive drug prescribed predominantly for prophylaxis of organ rejection post-transplant [22]. The gut microbiota contributes to complex metabolic interactions by its impact on drug metabolism, affecting the efficacy, toxicity, and bioavailability [23], contributing to high interindividual variability in drug metabolism and responses [24–27]. For example, *Faecalibacterium prausnitzii* directly metabolizes tacrolimus into less potent metabolites in vitro [28, 29]. The gut microbiota can reactivate the inactive form of a potent immunosuppressive agent mycophenolate mofetil and influence its pharmacokinetics [30, 31]. Immunosuppressants also significantly altered the gut microbiome [9, 32]. High doses of tacrolimus significantly altered the composition and structure of the gut microbiome [33].

Immunosuppressive drugs are always combined clinically with antibiotic regimens, heightening the complexity of interactions with the microbiota [34, 35]. The antibiotics are typically administered to transplant recipients to prevent or treat infection. However, antibiotics disrupt the gut microbiota, leading to reduced microbial diversity and an increased risk of infection from opportunistic pathogens [36, 37]. Given the consequences of modifying the gut microbiota on inflammation, immunity, and metabolism, sophisticated analyses that seek to identify the major variables, their interactions, and their effects are required to advance our understanding of the role of gut microbiota and their metabolic activities in immunosuppressed environments.

The goal of this study is to delineate early changes in metabolite profiles caused by immunosuppressive treatment, and its intricate interactions with the gut microbiome. We employed metagenomic, metabolomic and immunological approaches to compare the effects of tacrolimus and antibiotics on the gut lumen and circulation in a murine model. This model employs low-dose daily tacrolimus administration that effectively mirrors both clinical dosing and clinical events reminiscent of human pathology, surpassing models based on acute, binary measures of rejection [38, 39]. Distinct metabolic phenotype, or “metabotype” that constitute one or a set of compounds that reflect treatment effects [40], were elicited by tacrolimus. Distinct metabotypes after two days and seven days of treatment demonstrated significant and incremental effects imposed by continuous immunosuppressant treatment. Gut microbial community and composition was also persistently altered at these two time points. Though integrative analyses, our study underscored the prompt influence of immunosuppressive drugs on host metabolism in both gut and circulation, occurring ahead of detectable significant shifts in gut microbiota composition and structure. These rapid and sensitive metabolic signatures can be used as antecedent biomarkers to indicate the onset and progression of metabolic changes, highlighting their value as diagnostic and potential auxiliary therapeutic targets for managing metabolic disorders from prolonged use of immunosuppressants.

## Results

### Short-term tacrolimus-induced modest versus antibiotics-induced strong changes in the gut microbiome

**Fig. 1:**
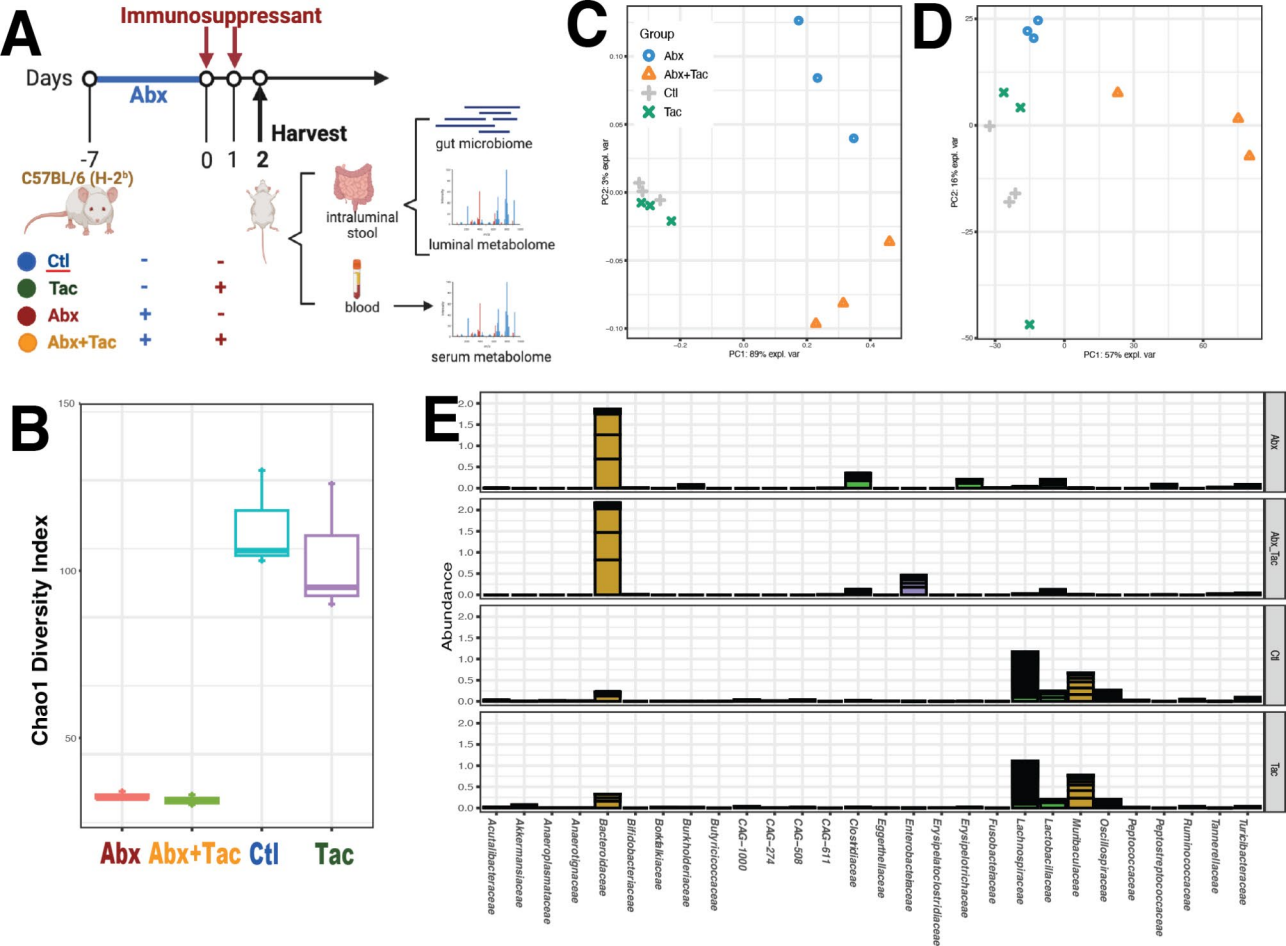
Short-term drug effect on gut microbiome. **A**) Experimental design. C57BL/6 mice were treated with antibiotics for 6 days only from day 7 to 1, tacrolimus 2 days only, the combination of antibiotics from day 7 to 1 followed by tacrolimus on day 1 and 2, or untreated no drug control. **B**) Chao1 diversity index (within-community diversity) of the four experimental groups. Canonical Correspondence Analysis (CCA) to demonstrate *de novo* clustering of gut microbiome **C**) taxonomic groups and **D**) microbial functional pathways characterized using HUMAnN2 (v0.11.2) [41] and Uniref90 database [42] based on Bray-Curtis distance. **E**) Cumulative relative abundance of bacteria groups in families for the four experimental groups, using ward linkage clustering based on Euclidian distance. *Abbreviations*: Abx: antibiotics; Ctl: no treatment control; Tac: tacrolimus; Abx + Tac: antibiotics with tacrolimus treatment

We first investigated the short-term responses (experimental design in Fig. 1A) to tacrolimus and antibiotics on the gut microbiome and metabolism. C57BL/6 mice were treated with antibiotics for 6 days only, tacrolimus for 2 days only, the combination of antibiotics followed by tacrolimus, or untreated no drug control. Whole community metagenomic sequencing of colon intraluminal fecal contents yielded 39.4 ± 8.0 (mean ± s.d.) million reads per sample after quality assessment (Supplemental Table 1A). Intraluminal fecal content from the jejunum showed that the majority (> 98%) of the reads were from the host (Supplemental Table 1B). Thus, the intraluminal content of the colon was used in subsequent analyses. Taxonomic composition using the comprehensive mouse gut metagenome catalog (CMGM) [43] showed 222 taxonomic groups at species level (Supplemental Table 1 C) in 64 genera (Supplemental Table 1D). Functional characterization using HUMAnN2 (Human Microbiome Project Unified Metabolic Analysis Network) (v0.11.2) [41] to determine the prevalence and abundance of metabolic functional units is presented in Supplemental Table 1E.

The effects of antibiotics and tacrolimus on gut microbiota were distinct. Antibiotic treatment alone or in combination with tacrolimus significantly reduced gut microbiota diversity (Fig. 1B) and altered the taxonomic composition and structure (Fig. 1C) as well as the functional makeup (Fig. 1D). Compared to tacrolimus alone or the untreated control, the antibiotic effect was much stronger with phylogenetic collateral sensitivity, as taxa from the same phylogenetic groups were simultaneously affected (Supplemental Fig. 1A). The most striking changes were in observed in Firmicutes (aka. Bacillota) with members from the families of Lachnospiraceae (Firmicutes), Oscillospiraceae (Firmicutes), Ruminococceae (Firmicutes), and Muribaculaceae (aka. S24-7, Bacteroidota) being depleted (Supplemental Fig. 1B–E). Antibiotics overpowered the effects of tacrolimus on the gut microbiome when used in combination (Fig. 1E). The most differentially abundant group was Enterobacteriaceae (*Enterobacter* and *Klebsiella pneumoniae*) for tacrolimus plus antibiotics, while the antibiotics-only treatment group had a few low abundant groups in Firmicutes and Burkholderiales (*Clostridium* and *Paeniclostridium sordellii*) (Supplemental Fig. 3). Otherwise, the antibiotics treated groups with and without tacrolimus were highly similar.

Unlike antibiotics, tacrolimus alone induced only modest changes in gut microbiota, presenting high similarities to the control in community diversity, taxonomic composition and structure, and functional makeup (Fig. 1B–E). The most affected taxa were in low abundance and sporadically distributed in different taxonomic groups without relation to the phylogenetic range (Supplemental Fig. 3). *Akkermansia muciniphila* (Verrucomicrobiae) was more abundant in the tacrolimus group, whereas a few low abundant Clostridia taxa were more abundant in the control group (Supplemental Fig. 2). Overall, antibiotic treatment, with and without tacrolimus, strongly affected the gut microbiome, and this wide spectrum impact was related to the phylogenetic range. The 2-day tacrolimus treatment had modest effects on the gut microbiome, which was not related to the microbial phylogenetic range.

### Short-term tacrolimus treatment induced profound changes in metabolic activities in both gut lumen and serum

To investigate gut metabolism, we profiled the metabolome of paired intraluminal stool and serum samples using capillary electrophoresis-mass spectrometry (CE/MS) (Fig. 1A). After quality assessment, 247 luminal metabolites were included, out of which 233 were annotated by at least one reference from PubChem [44], Kyoto Encyclopedia of Genes and Genomes [KEGG, [45]], or the Human Metabolome Database [HMDB, [46]] (Supplemental Table 2 A). KEGG BRITE hierarchy remains the benchmark for function classification, enabling the allocation of a metabolite to its respective KEGG compound, functional module, involved pathways, and functional class. According to the KEGG BRITE hierarchical classification system, 84 luminal metabolites were assigned in this system. The most prevalent class of luminal metabolites was amino acid metabolism, comprising 40.5% (34/84) of all annotated metabolites (Supplemental Table 2 C). These metabolites belong to pathways in arginine and proline metabolism, arginine biosynthesis, cysteine and methionine metabolism, histidine metabolism, tryptophan metabolism, and glycine, serine and threonine metabolism. Together with other amino acid metabolites (i.e., β-alanine metabolism, glutathione metabolism), the amino acid metabolism-related metabolites comprised 48.7% of the total luminal metabolome (Supplemental Table 2D). Other prevalent classes included carbohydrate metabolism (14.3%, 12/84), nucleotide metabolism (10.7%, 9/84), lipid metabolism (9.5%, 8/84), metabolism of cofactors and vitamins (8.3%, 7/84), and the biosynthesis of other secondary metabolites (3.6%, 3/84). Individual metabolites were characterized in 111 functional modules such as polyamine biosynthesis (arginine = > agmatine = > putrescine = > spermidine) to indicate key metabolic processes (Supplemental Table 2E).

The serum metabolome was estimated to be approximately 80% similar to the paired luminal metabolome based on KEGG functional modules. A total of 262 serum metabolites were included after quality assessment, of which 233 were annotated (Supplemental Table 2B). 89 serum metabolites were assigned to the KEGG BRITE hierarchical classification system. The most prevalent class was amino acids metabolism (42.7%, 38/89), an even higher proportion than the luminal metabolome. Lipid metabolism (12.4%, 11/89) and xenobiotics biodegradation and metabolism (3.4%, 3/89) were also higher in the serum. Conversely, carbohydrate (12.4%, 11/89) and nucleotide metabolism (7.9%, 7/89) were higher in the lumen. Metabolic pathways were also similar in the lumen and serum. The main pathways for which the lumen metabolome had more coverage included protein digestion and absorption, biosynthesis of cofactors, taurine and hypotaurine metabolism, glutathione metabolism, neuroactive ligand-receptor interaction, cysteine and methionine metabolism, purine metabolism, and the cAMP signaling pathway. Conversely, the serum metabolome had higher coverage of lysine degradation, phenylalanine metabolism, tryptophan metabolism, fatty acid biosynthesis, tyrosine metabolism, and glycine, serine, and threonine metabolism. Approximately 80% of the serum functional modules shared key metabolic processes with the lumen (Supplemental Table 2E–G). The rest were either present in serum or gut. For instance, ornithine biosynthesis (glutamate = > ornithine) was present in lumen but not in serum.

**Fig. 2:**
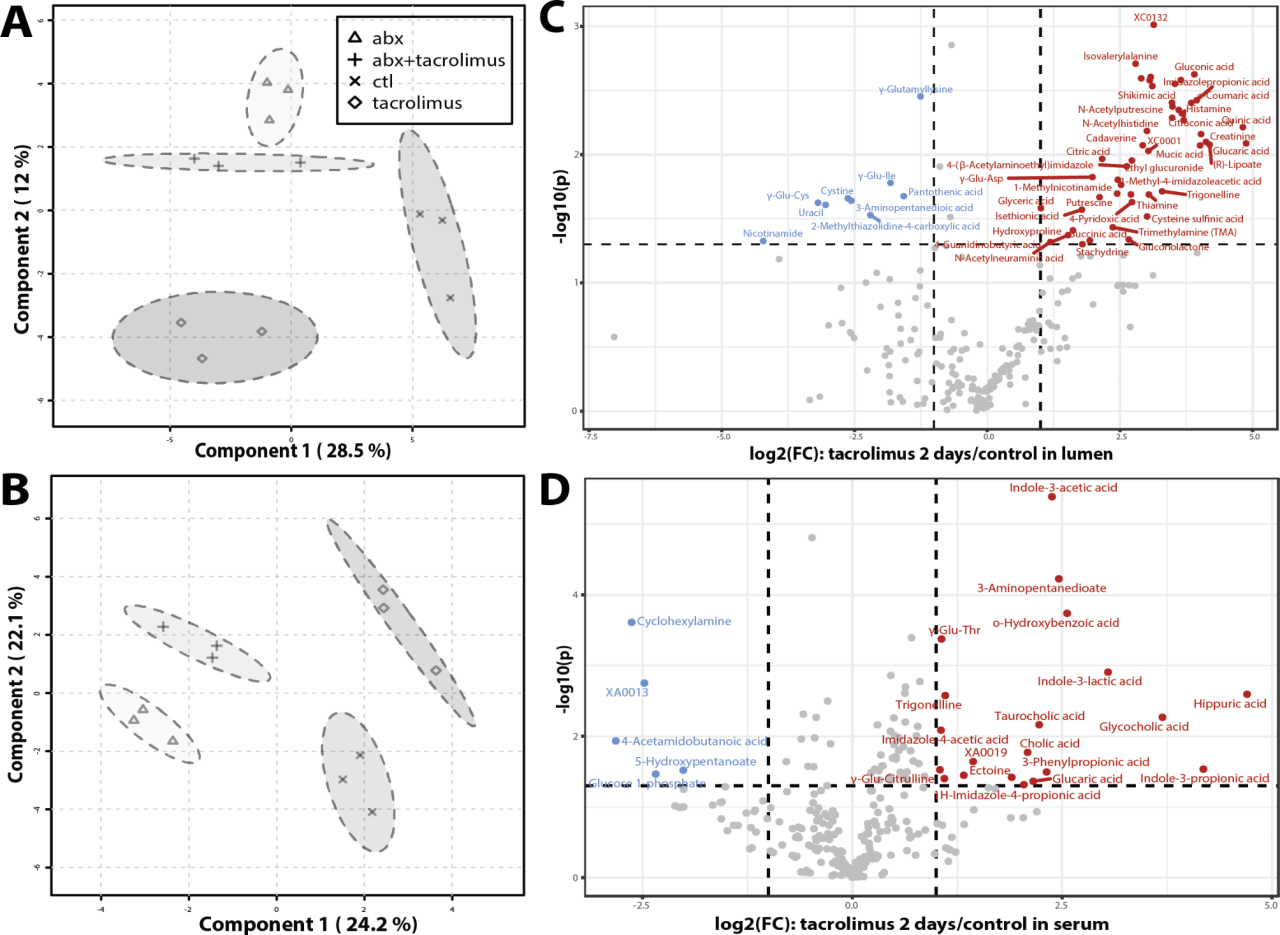
Short-term drug effect on metabolome. Clustering of **A**) luminal metabolites and **B**) serum metabolites using sparse Partial Least Squares Discriminant Analysis (sPLS-DA) to demonstrate metabolic phenotypes due to drug treatments. Volcano plot combines results from fold change (FC) analysis to show significantly increased metabolites after 2-day tacrolimus treatment in **C**) lumen or **D**) serum. A metabolite is shown if FC is > 2 and p value is < 0.05 based on 2-sample t-test. Original metabolite measurement without normalization was used in FC analysis

Tacrolimus elicited distinct and strong metabolic changes within 2 days of treatment. Sparse Partial Least-Squares Discriminant Analysis (sPLS-DA) was employed to analyze the large dimensional datasets that had more variables (metabolites) than samples (p > > n) to produce robust and easy-to-interpret models [47]. Distinct metabolic profiles after 2-day tacrolimus treatment were observed in both lumen (Fig. 2A) and serum (Fig. 2B), indicating the significant impact of tacrolimus on the metabolism of both the circulation and gut lumen. The key metabolites that contributed to the distinction among treatment groups are shown in Supplemental Fig. 4. These compounds offer a comprehensive insight into the treatment-induced metabolic shifts. An overview of the most differentially abundant compounds among the treatment groups is shown in hierarchical clustering heatmaps in Supplemental Fig. 5. Tacrolimus elicited stronger metabolic changes than antibiotics in terms of the number of metabolites and pathways that were affected. Comparison with antibiotics revealed 10 times more significantly induced luminal metabolites and 4 times more serum metabolites elicited by tacrolimus (Fig. 2C and D). Comparison with the no treatment control revealed > 5 times more significantly increased luminal metabolites and 4 times more serum metabolites in tacrolimus than in antibiotics (Supplemental Table 3 A, 3B). Pathway enrichment analysis also revealed that more pathways were significantly affected by tacrolimus in both the lumen and serum, which was evaluated from the dimensions of pathway topology (i.e., more hits observed in the pathway or more influential “hub” hits) and pathway significance (i.e., more compounds with statistical significance) (Supplemental Table 3 C, 3D). Compared to the no treatment control, tacrolimus significantly induced luminal pathways in vitamin B6 metabolism, arginine and proline metabolism, histidine metabolism, glyoxylate and dicarboxylate metabolism, and nicotinate and nicotinamide metabolism. Compared to antibiotics, tacrolimus additionally induced luminal pathways in butanoate metabolism, alanine, aspartate and glutamate metabolism, cysteine and methionine metabolism, pantothenate and CoA biosynthesis, β-alanine metabolism, arginine biosynthesis, and starch and sucrose metabolism. Compared to the no treatment control, tacrolimus significantly increased serum metabolic pathways in alanine, aspartate and glutamate metabolism, glyoxylate and dicarboxylate metabolism, tryptophan indole pathway, primary BA, taurine and hypotaurine, TCA, and alanine, aspartate and glutamate metabolism. Additional serum pathways induced by tacrolimus compared to antibiotics included nicotinate and nicotinamide, tryptophan metabolism of serotonin and L-kynurenine, D-glutamine and D-glutamate, and thiamine metabolism. Overall, tacrolimus exerted a stronger effect on both luminal and circulating metabolism of a selected pool of essential amino acid and carbohydrate metabolism pathways.

Unlike tacrolimus, antibiotics induced only modest changes in the metabolome. The most elevated luminal compounds were primary BAs (Supplemental Fig. 6A). Serum levels of a few compounds were elevated, including the antibiotic itself (i.e., metronidazole) (Supplemental Fig. 6B). Serum pathways of alanine, aspartate, and glutamate metabolism, arginine and proline metabolism, arginine biosynthesis, and valine, leucine and isoleucine biosynthesis were increased in antibiotics. However, most of these pathways decreased in the lumen after antibiotic treatment. The combined treatment of tacrolimus and antibiotics did not amplify the number of affected luminal metabolic pathways in comparison to the influence of antibiotics alone (Supplemental Table 3 C). However, it did manifest changes in serum metabolic pathways, notably in the metabolism of taurine, hypotaurine, primary bile acid biosynthesis, and histidine (Supplemental Table 3D). When comparing the effects of the combination of tacrolimus and antibiotics with tacrolimus alone, there were evident alterations in the gut lumen pathways related to butanoate metabolism, and the metabolism of alanine, aspartate, and glutamate. Furthermore, the combined treatment impacted pathways concerning the metabolism of lysine, valine, leucine, isoleucine, and glutathione. These observations underscore the distinct metabolic shifts induced by antibiotics and tacrolimus, emphasizing their unique and combined effects on host metabolism.

### Tacrolimus exerted additional effects on gut microbiome and metabolism after prolonged administration

**Fig. 3:**
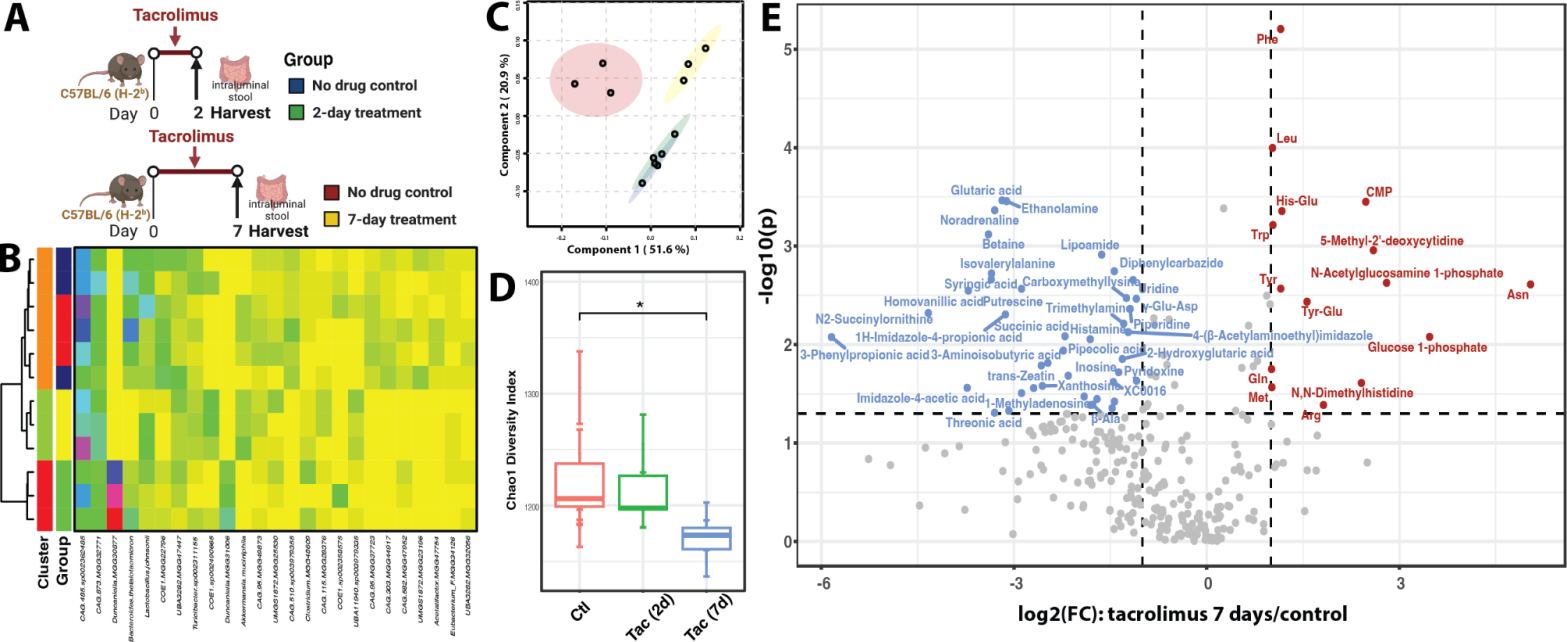
Gut microbiome and luminal metabolome after tacrolimus treatment for 2 or 7 days. **A**) Experimental design. Colors indicate different treatment groups. **B**) Heatmap of the top 25 most abundant intestinal bacterial taxa. Ward linkage clustering used to cluster samples based on the Jensen-Shannon distance calculated in vegan package in R [48]. **C**) PLS-DA to demonstrate clusters by treatment groups. Circles indicate 95% confidence region of each group. **D**) Chao1 diversity index of experimental groups. Wilcoxon test to estimate the significance value. * denotes significance value < 0.05. **E**) Volcano plot combines results from FC analysis to show significantly increased metabolites after 7-day tacrolimus treatment comparing to its respective no treatment control. A metabolite is shown if FC is > 2 and p value is < 0.05 based on 2-sample t-tests. Original metabolite measurement without normalization in FC analysis

Seven-day tacrolimus treatment was investigated to characterize the impact of prolonged tacrolimus use (experimental design in Fig. 3A, Supplemental Table 4 A). The gut microbiota of the 2- and 7-day untreated controls were clustered together, distinct from the 2- and 7-day treatment groups, as shown in the heatmap (Fig. 3B). Using principal component analysis based on taxonomic composition and structure (Fig. 3C), we observed a distinct separation between the mice group that received 7 days of tacrolimus treatment and its control compared to the group that received only 2 days of treatment with its control. Therefore, the more pronounced separation seen in the 7-day treatment group suggests that the prolonged exposure to tacrolimus has a more robust effect, when all groups are analyzed on a uniform scale. Seven-day tacrolimus treatment was more effective in terms of the number of differentially abundant taxa (Supplemental Fig. 7A) and significantly reduced diversity of the gut microbiota (Fig. 3D). Compared with the 2-day tacrolimus treatment, the most significantly altered taxonomic groups after 7-day treatment were distributed in a wider phylogenetic range, including Clostridiales, Verrucomincrobiota, and Saccharimonadales (Supplemental Fig. 7B). Overall, these results indicate that tacrolimus reduced commensals and overall diversity and this effect was more pronounced with longer administration.

Metabolome analyses of intraluminal stool revealed 213 metabolites after 2- and 7-day treatments **(**Supplemental Table 4B, 4 C), comprising 88.8% of the 2-day metabolome and 70.3% of the 7-day metabolome. The remaining 11.2% and 29.7% were detected only in the 2- and 7-day treatments, respectively. A distinct set of significantly increased metabolites was observed after 7 days of tacrolimus treatment compared to no drug control, including sets of amino acids (Phe, Leu, Trp, Tyr, Gln, Met, Arg, Asn) and dipeptides (His-Glu, Tyr-Glu) (Fig. 3E). Multiple metabolites that were significantly reduced after seven days but increased in the 2-day treatment group included isovalerylalanine, putrescine, λ-Glu-Asp (L-Glutamyl-L-aspartic acid), trimethylamine (TMA), succinic acid, histamine (histidine pathway), and threonic acid. Taking together, these results show that the effects of tacrolimus on the gut microbiota and metabolome are not immediate and accrue over time.

### Modularity of gut microbiota and metabolome indicates network effects due to drug treatment

**Fig. 4:**
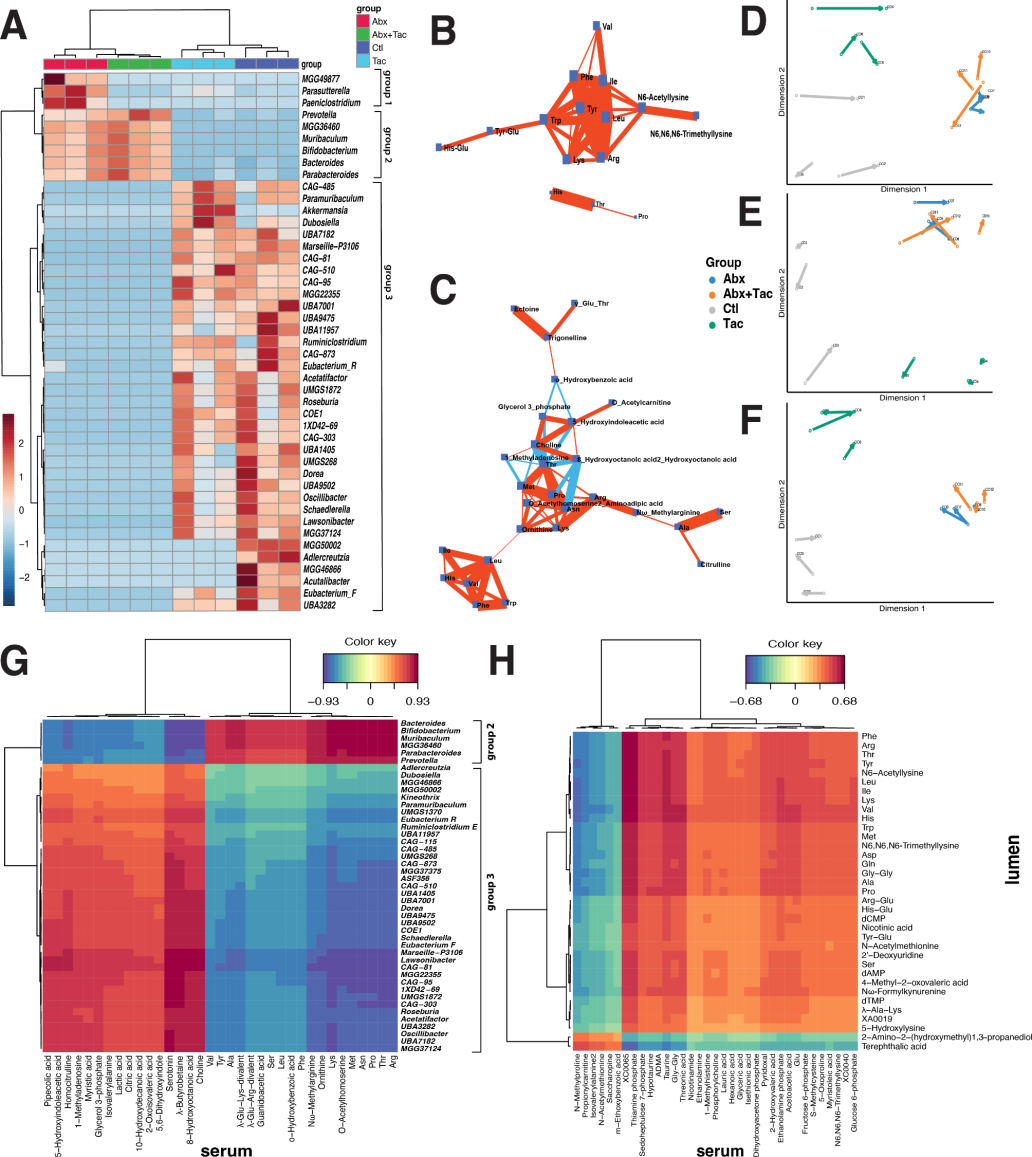
Correlations between luminal and circulating metabolome. **A**) Hierarchical clustering heatmap of gut microbiota using genera. Sub-correlation network of **B**) luminal metabolome and **C**) serum metabolome. See Supplemental Fig. 8 for the rest of the network. Debiased Sparse Partial Correlation (DSPC) network was used [51]. Nodes denote taxonomic groups or metabolites; edges represent association measures. Default cutoff value was used for degree filter and betweenness. Correlation significance value < 0.01 used. Sparse partial least squares (or Projection to Latent Space, PLS) [52, 53] to integrate pairwise datasets of **D**) gut microbiome and luminal metabolome, **E**) gut microbiome and serum metabolome, and **F**) luminal metabolome and serum metabolome from paired samples of the same mouse. Samples represented in the latent space from multiple coordinates to demonstrate the level of agreement between the two paired data sets. Arrows connecting paired samples of the same mouse from the indicated two datasets. Cluster Image Map of the Pearson correlation coefficients between two matched datasets [54] of **G**) gut microbiota and serum metabolome and **H**) lumen metabolome and serum metabolome. Hierarchical clustering applied on the rows and columns of the similarity matrix simultaneously. The color represents the values of the similarity matrix when performing two dataset integration. Ward linkage to cluster both samples and metabolites based on their Euclidean distance. Color bar indicates the scaled z-score of each feature

Microbes and metabolites do not operate in isolation but interact with each other to maintain homeostasis, commonly referred as network effect [49]. The group of microbes or metabolites that operate together, indicated as in statistically significant positive or negative corrections. These correlations reflect the tendency of these entities to increase or decrease in concert and may suggest a shared role in particular biological processes or responses. The concept of modularity was thus used to reflect the degree of node connectivity to which a network can be divided into subgroups or modules to understand the organization and functional relationships within a complex system [50]. Highly connected components often have similar functions or are part of the same biological process in response to different stimuli. High modularity was observed in the gut microbiota and the luminal and serum metabolome in our datasets. The gut microbiota was *de novo* clustered into three distinct clusters (Fig. 4A): cluster 1 was enriched in antibiotics only; cluster 2 was elevated in antibiotics only or with tacrolimus that contained taxa such as *Prevotella*, *Bacteroides*, *Muribaculum*, and *Bifidobacterium*, which included a large number of taxa enriched in either tacrolimus or no drug control, such as *Roseburia, Oscillibactera, CAG-81*, *Acetatifactor*, *Lawsonibacter*, and *Schaedlerella*. Taxa within clusters 2 and 3 exhibited distinct inter-relationships. Specifically, taxa in cluster 2, including *MGG36460, Muribaculum*, and *Bifidobacterium*, were inversely correlated with *Marseille-P3106* and *CAG-81* from cluster 3, as visualized in the correlation network in Supplemental Fig. 8A. Moreover, *Bacteroides* from cluster 2 showed a negative correlation with *UBA3282* and *ASF356* of cluster 3. In contrast, the remaining taxa within cluster 3 displayed positive correlations amongst themselves. These results suggest concerted changes among subsets of the gut microbial community in response to different treatments.

Luminal metabolites also formed networks that contained compounds that were either positively or negatively correlated, suggesting concerted responses to different treatments. Four amino acid and derivative networks were observed and were positively correlated within-network, as shown in Supplemental Fig. 8B: (i) Arg, Trp, Val, Ile, Phe, Tyr, Leu, Lys, Tyr-Glu, His-Glu, N6-acetyllysine, and N6, N6, N6-trimethyllysine (Fig. 4B). The majority of these amino acids also exhibit significantly elevated levels in the day-7 tacrolimus treatment group compared to control, as shown in Fig. 3E. These results collectively suggest a coordinated modulation of a set of amino acids in response to immunosuppressant; (ii) His, Thr, and Pro; (iii) γ-glutamyl dipeptides γ-Glu-Gln, γ-Glu-Trp, γ-Glu-Met and γ-Glu-Ala; and iv) Ser-Glu, Glu-Glu, Ile-Pro-Pro, and Val-Pro-Pro, which were negatively correlated with amino acid derivatives of carnitine, creatine, and betaine. In addition to amino acids, other networks were found to be functionally related to carbohydrates and nucleosides and nucleotides metabolism (data not shown).

Serum metabolites are also highly modular. Multiple correlation networks were observed (Supplemental Fig. 8C): (i) purine metabolism; (ii) pentose phosphate pathway; (iii) amino acid metabolism that includes Arg, Lys, Pro, Met, Thr, Ala, and Ser and Nω-methyarginine, citrulline, and ornithine (Fig. 4C), many of which were enriched in antibiotics and tacrolimus combined treatment group (Supplemental Fig. 4B); and (iv) amino acid derivatives (isovalerylalanine, 1 N-acetylleucine, 1 H-Imidazole-4-propionic acid, 3-phenylpropionic acid, N-acetylphenylalanine) that were negatively correlated with glycerophospholipid ethanolamine phosphate. Overall, this result indicates coordinated changes in metabolic pathways among functionally related components between the gut and systemic metabolites across all mice groups post-treatment. The shifts seen in metabolic pathways of the gut and systemic circulation are not uniform across groups. Instead, they exhibit distinct patterns depending on the type of treatment, which suggests different drug treatments invoke distinct biological processes that shape the gut ecosystem and influence systemic metabolism. Our results offer a granular look into the interactions and responses elicited by different treatments, to understand the influence of the gut microbiome on systemic metabolism in the immune suppressed environment.

### Highly correlated gut and systemic metabolism

Sparse partial least squares (or projection to Latent Space, PLS) was employed to represent paired gut microbiota and luminal metabolome of the same mouse in the same latent space to demonstrate their level of agreement [52, 53]. The metabolic phenotype in the tacrolimus or antibiotic groups produced more “homogeneous” sample projections, as depicted by the short average arrow length between the paired gut microbiota and luminal metabolome (Fig. 4D), luminal and serum metabolome (Fig. 4E), gut microbiome, and serum metabolome (Fig. 4F).

The network modules of the gut microbiota and metabolome were correlated. Microbiota cluster 2 was positively correlated with luminal metabolites belonging to the carbohydrate metabolism pathway (glucaric acid, gluconic acid, 6,8-thioctic acid, quinic acid, gluconolactone, and creatinine) (Supplemental Fig. 8D), as well as with serum amino acids (Val, Tyr, Ala, Leu, Ser, Phe, Lys, Met, Asn, Pro, Thr and Arg) (Fig. 4G), many of which were enriched in antibiotics only or antibiotics with tacrolimus (Supplemental Fig. 5B). Microbiota cluster 3 was positively correlated with luminal metabolites of S-adenosylmethionine (SAM), GABA, glyceric acid, glycine, symmetric dimethylarginine (SDMA), asymmetric dimethylarginine (ADMA), spermidine, N1-acetylspermidine, citrulline, and O-acetylcarnitine (Fig. 4G). These metabolites are essential compounds in interconnected pathways of arginine metabolism, polyamine metabolism, nitric oxide regulation, and urea cycle. Microbiota cluster 3 was also positively correlated with the serum metabolites of serotonin (5-HT), 5-hydroxyindoleacetic acid (5-HIAA), lactate acid, glycerol 3-phosphate, Nω-methylarginine, butyrobetaine, and choline (Fig. 4H), which were within the same network (Supplemental Fig. 8C). Furthermore, the luminal amino acid network correlated with serum metabolites involved in glucose homeostasis, nitric oxide regulation, and BA metabolism (Fig. 4F). These results indicate that the correlations between gut and systemic metabolism occur through interconnected pathways, particularly amino acid metabolism.

### Altered amino acid metabolism in an immune suppressed environment

Since tacrolimus elicited distinct metabolic phenotypes, we sought to define the metabolic phenotype, or “metabotype”, which reflects one or a set of compounds that inform about the treatment effect [40]. Metabolites from the functional pathways most induced by tacrolimus were investigated. In particular, we used the ratio of two metabolites that were either directly linked or shared a common precursor in a pathway. The ratio is less subject to individual variations and is more reflective of the dynamic changes in metabolic fluxes or shifts compared to the absolute concentration of a single metabolite [55].

**Fig. 5:**
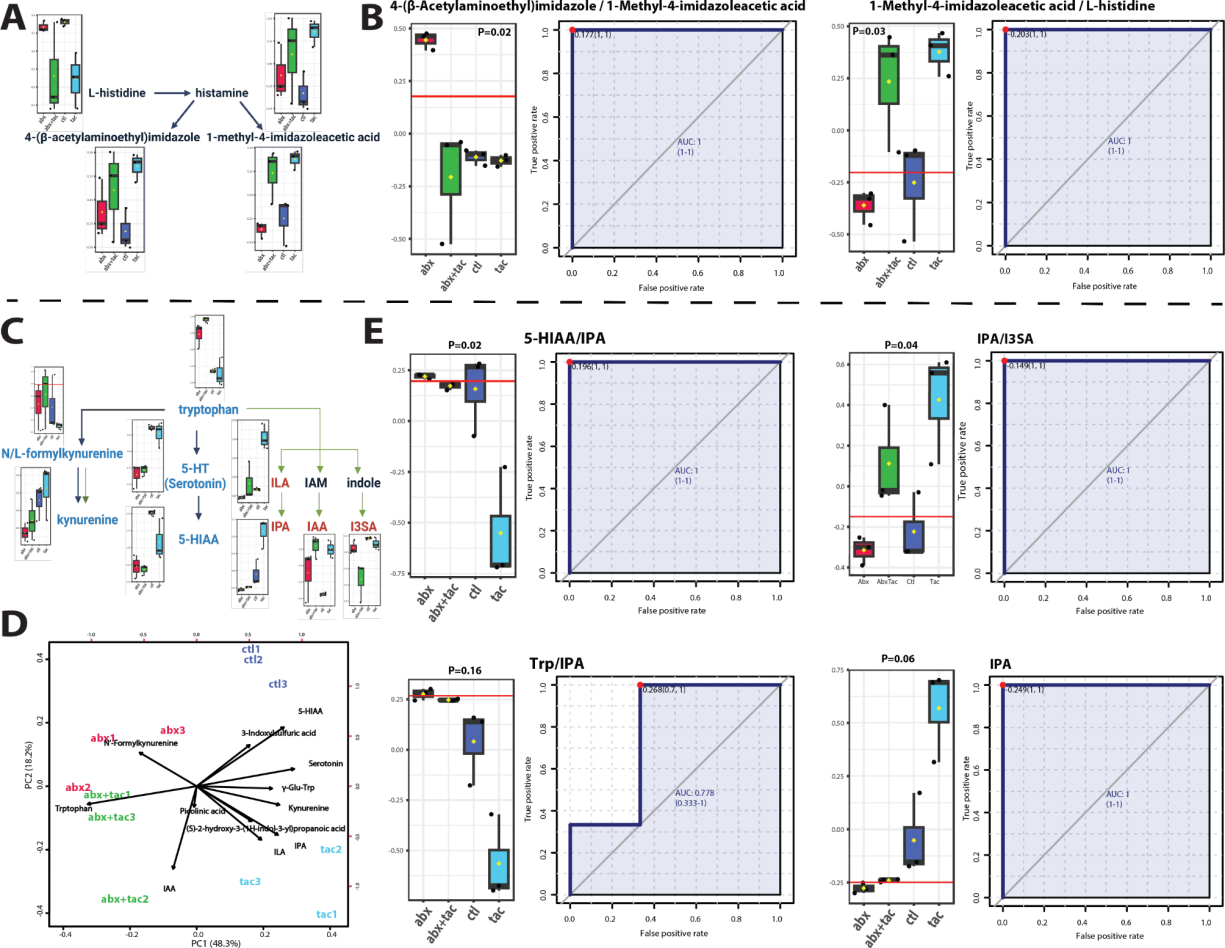
Histidine and tryptophan metabolism after 2-day drug treatment. **A**) Pathway of histidine metabolism to histamine with the metabolite products of 1-methyl-4-imidazoleacetic acid and 4-(β-acetylaminoethyl)imidazole. Illustration adapted from KEGG histidine metabolism pathway (map00340) [45]. **B**) Metabolite conversion ratio of 4-(β-acetylaminoethyl)imidazole to 1-methyl-4-imidazoleacetic acid, and of 1-methyl-4-imidazoleacetic acid to histidine in the four 2-day treatment groups of tacrolimus, antibiotics, tacrolimus and antibiotics together, and no treatment control. **C**) Tryptophan metabolism, including the kynurenine pathway, indole pyruvate pathway, and serotonin pathways. Illustration adapted from KEGG histidine metabolism pathway (map00380) [45]. **D**) Biplot of PCA of tryptophan metabolism pathway. Loading vectors and principal components labeled. **E**) IPA concentration and metabolite conversion ratio of 5-HIAA (5-hydroxyindoleacetic acid) to indole-3-propionic acid (IPA), IPA to 3-indoxylsulfuric acid (I3SA), tryptophan to IPA in the four 2-day treatment groups of tacrolimus, antibiotics, tacrolimus and antibiotics together, and no treatment control. Y axis represents the ratios normalized level between the indicated metabolites. The ROC curve used to calculate area under curve (AUC) as a metric quantifying the overall ability of the metabolite to correctly classify the experimental conditions. Closest to top-left core of ROC (red dot) as the optimal cutoff value, shown in bargraph (red line). P value was calculated using 2-sample t-tests between tacrolimus and control groups and displayed atop the boxplots. Black points representing mice, horizontal line representing mean, yellow dots presenting median, the top and bottom of the box are the lower and upper quartile

**Fig. 6:**
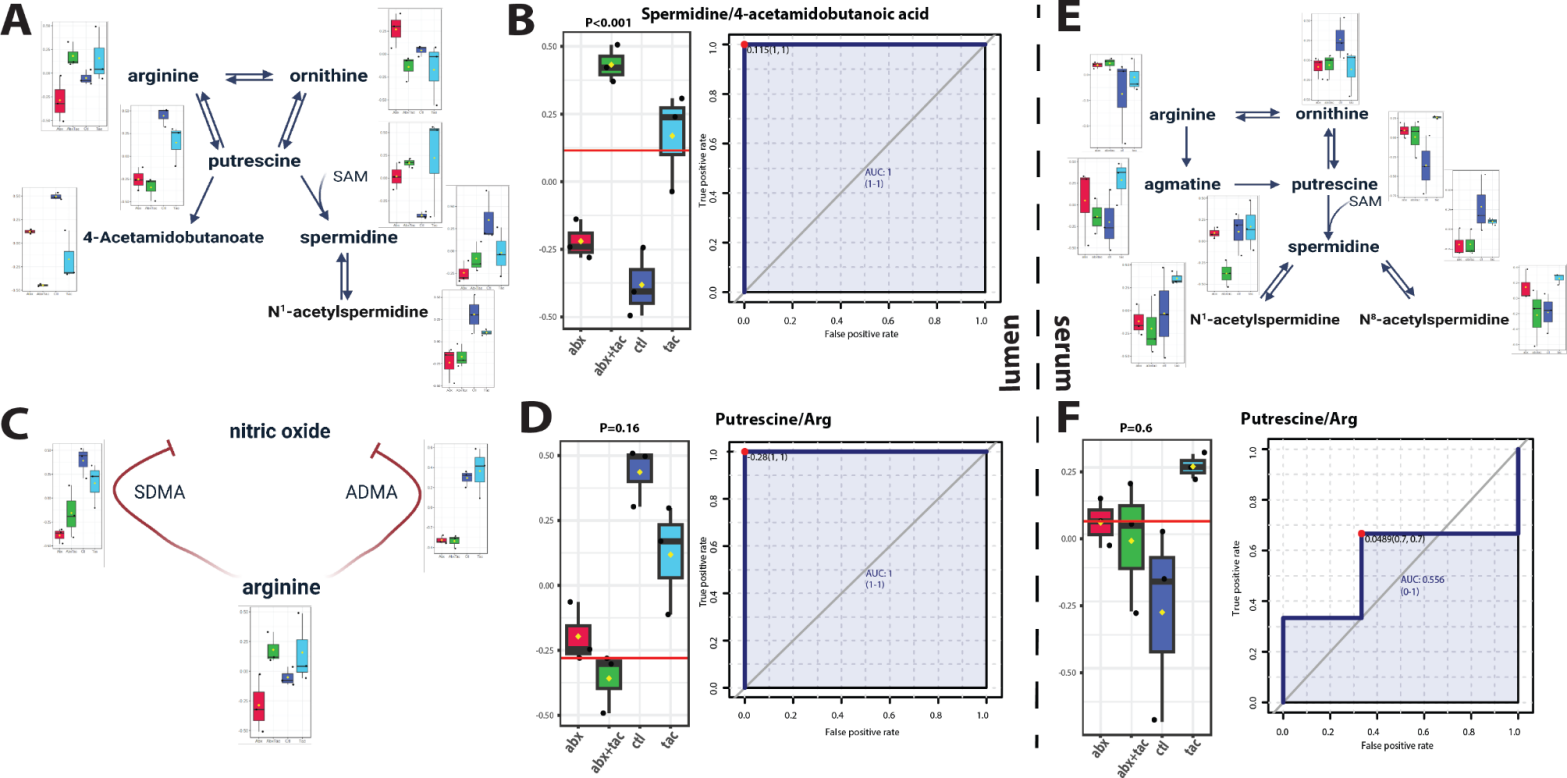
Arginine and polyamine metabolism after 2-day drug treatment. Pathway of arginine metabolism toward polyamine biosynthesis and metabolism in **A**) serum and **E**) gut lumen, and **C**) pathway of arginine metabolized to nitric oxide synthesis. Illustration adapted from KEGG histidine metabolism pathway (map00330) [45]. Metabolite conversion ratio plot in **B**) spermidine to 4-acetamidobutanoid acid in serum, putrescine to arginine in both **D**) lumen and **F**) serum in the four 2-day treatment groups of tacrolimus, antibiotics, tacrolimus and antibiotics together, and no treatment control. Y axis represents the ratios normalized level between the indicated metabolites. The ROC curve used to calculate area under curve (AUC) as a metric quantifying the overall ability of the metabolite to correctly classify the experimental conditions. Closest to top-left core of ROC (red dot) as the optimal cutoff value, shown in bargraph (red line). P value was calculated using 2-sample t-tests between tacrolimus and control groups and displayed atop the boxplots. Black points representing mice, horizontal line representing mean, yellow dots presenting median, the top and bottom of the box are the lower and upper quartile. *Abbreviations*: ADMA: asymmetric dimethylarginine; SDMA: symmetric dimethylarginine; SAM: S-adenosylmethionine

**Fig. 7:**
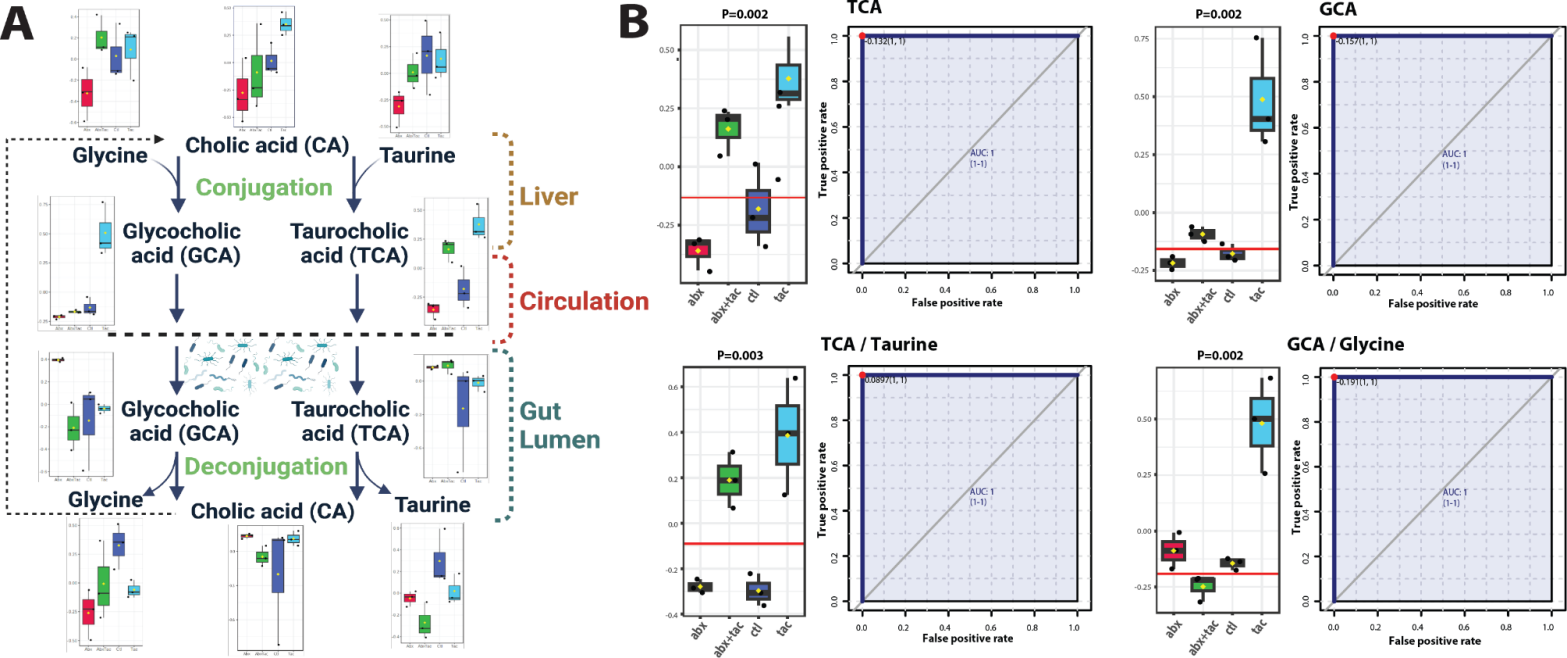
Primary bile acids conjugation changes after 2-day drug treatment. **A**) Pathway of bile acid conjugation in live, serum and gut lumen. Illustration adapted from KEGG primary bile acid biosynthesis pathway (map00120) [45]. **B**) Metabolite TCA and GCA concentration in serum and conversion ratio of taurine to TCA and of GCA to glycine. The ROC curve used to calculate area under curve (AUC) as a metric quantifying the overall ability of the metabolite to correctly classify the experimental conditions. Closest to top-left core of ROC (red dot) as the optimal cutoff value, shown in bargraph (red line). P value was calculated using 2-sample t-tests between tacrolimus and control groups and displayed atop the boxplots. Black points representing mice, horizontal line representing mean, yellow dots presenting median, the top and bottom of the box are the lower and upper quartile. *Abbreviations*: CA: cholic acid; GCA: glycocholic acid; TCA: taurocholic acid

Amino acid metabolic pathways, including histidine, tryptophan and arginine metabolism, were the most prominent among the compounds most significantly affected by tacrolimus (Figs. 5, 6 and 7). There was increased conversion of histidine to histamine and then to 1-methyl-4-imidazoleacetic acid, instead of conversion to 4-(β-acetylaminoethyl)imidazole (Fig. 5A and B), suggesting that the metabolism of histidine in the lumen is upregulated by tacrolimus. Three tryptophan metabolism pathways were observed in the serum, including the kynurenine, indole pyruvate, and serotonin pathways (Fig. 5C). The indole and serotonin pathways were significantly elevated by tacrolimus and/or reduced by antibiotics (Fig. 5D). The ratio of substrates involved in the indole pyruvate pathway, in which tryptophan is converted to indole-3-propionic acid (IPA) or ILA, was highest in the tacrolimus group (Fig. 5E). Serotonin (5-HT, 5-hydroxytryptamine) were also elevated by tacrolimus and/or reduced by antibiotics. Since the indole pathway requires microbial metabolism [56], while serotonin is primarily produced in the enterochromaffin cells of the gastrointestinal tract and released into the bloodstream [57], our results indicated that tryptophan metabolism in response to tacrolimus included synergistic reactions by the gut microbiome and intestinal epithelia that together directed the enzymatic reactions in tryptophan metabolism.

We further investigated the hydroxylated form of amino acids; hydroxylation is a post-translational modification that significantly influences protein structure and function, subsequently influencing immune responses [58, 59]. There were 20 hydroxylated serum metabolites and 9 fecal hydroxylated metabolites. The serum hydroxylated metabolites were defined by treatment (Supplemental Fig. 9A), indicating distinct modifications under treatment conditions. Tacrolimus increased 2-hydroxyglutaric acid (Supplemental Fig. 9B), an “oncometabolite”, and its accumulation promotes the formation and progression of cancer [60]. In the antibiotic-only group, *p*-hydroxyphenylpyruvic acid and 2-hydroxybutryic acid were elevated (Supplemental Fig. 9B). Antibiotics with tacrolimus distinctively increased the levels of hydroxyproline and decreased the levels of 3-(4-hydroxyphenyl)propionic acid, 5-HIAA (5-hydroxyindoleacetic acid), 5,6-dihydroxyindole, 5-hydroxypentanoic acid, and 3-hydroxybutyric acid. Metabolites such as 2-hydroxyglutaric acid, hydroxyproline, and 5-HIAA are known immune regulators, and others such as 2-hydroxybutryic acid and 3-(4-hydroxyphenyl)propionic acid are known metabolites derived from gut microbiota, indicating the potential impact of treatments on disrupted microbiota, metabolite production, and immune responses.

### Tacrolimus induces augmented polyamine metabolism

Tacrolimus induced distinct changes in arginine metabolism in both the lumen and serum (Fig. 6A). In serum, there was increased putrescine accompanied by an increased arginine to putrescine conversion ratio (Fig. 6B). The level of 4-acetamidobutanoate was significantly decreased, accompanied by a significantly increased ratio of spermidine to 4-acetamidobutanoate, indicating the directed metabolism by tacrolimus from arginine to putrescine, followed by spermidine and N^1^-acetylspermidine in the circulation (Fig. 6D). The luminal arginine metabolic pathway was also active (Fig. 6E). This was supported by the increased putrescine to arginine ratio (Fig. 6F), indicating directed metabolism by tacrolimus from arginine to putrescine, and then to N^1^-acetylspermidine and N^8^-acetylspermidine in the gut lumen. Together, these results suggest that tacrolimus drives active arginine metabolism that is directed towards polyamine metabolism in both lumen and circulation.

Arginase I and nitric oxide (NO) synthase (NOS) compete for arginine to produce either polyamines or NO [61]. Potent NOS inhibitors including asymmetric dimethylarginine (ADMA) and its enantiomer symmetric dimethylarginine (SDMA) [62–65], are significantly higher in tacrolimus group compared to the group treated with antibiotics (Fig. 6C). This suggests a potential inhibition of NOS by tacrolimus, and/or a diminished NOS inhibition in the presence of antibiotic administration. S-adenosylmethionine (SAM) is involved in the methylation of arginine to form ADMA [66], and SAM levels were increased by tacrolimus (Fig. 6A), supporting the notion that increased SAM levels lead to the formation of more ADMA, which in turn inhibited NOS activity. Together, these results demonstrate the diverted metabolism from arginine towards increased polyamine biosynthesis by tacrolimus, reflecting an increased requirement for cellular growth and proliferation in an immunosuppressed environment.

### Altered BA conjugation in gut lumen and circulation

BA and their conjugation processes are intricately associated with the mechanisms of immunosuppression. These connections are based on their multifaceted roles in mediating immune responses, influencing gut microbiome composition and function, and potentially affecting the metabolism of the immunosuppressant drugs [67]. BA conjugation is an essential process that occurs in the liver, where primary BAs, such as cholic acid (CA), are combined with amino acids, such as glycine or taurine, to form conjugated BAs of glycocholic acid (GCA) or taurocholic acid (TCA) (Fig. 7A). BAs are normally reabsorbed in the intestine and recycled back into the liver through enterohepatic circulation. The metabolic phenotypes of the primary BAs under different drug treatments were distinct (Fig. 7B). Elevated serum GCA and TCA levels were observed, accompanied by elevated GCA to glycine and TCA to taurine ratios by tacrolimus (Fig. 7B). Increased luminal CA to GCA and CA to TCA ratios by tacrolimus were also observed (data not shown), suggesting increased deconjugation of BAs in the lumen, a process known to be driven primarily by gut microbiota via bile salt hydrolase (BSH) enzymatic activities [68]. Antibiotic treatment caused significantly higher luminal GCA and TCA levels (Supplemental Fig. 9), indicating reduced deconjugation in the gut lumen. As shown above, antibiotics affected entire taxonomic groups of Firmicutes and Bacteroidota (Fig. 1E, Supplemental Fig. 1A), including the majority of identified BSH-containing bacteria such as *Blautia*, *Eubacterium*, *Clostridium*, *Lactobacillus*, and *Roseburia* [69, 70]. Together, these results indicate that antibiotic and tacrolimus treatments both disrupt BA homeostasis, but likely through different mechanisms.

### Microbiome-dependent metabolic activities

In addition to previous knowledge on microbe-derived metabolites [59], we performed *in silico* modeling to characterize microbial involvement in metabolic processes. To relate actual metabolite measurements to paired microbiome metabolic potentials (CMP), we calculated the set of metabolic reactions that each microbial taxon is predicted to be capable of performing using MIMOSA2 (Model-based Integration of Metabolite Observations and Species Abundances) [71]. The top metabolites correlated with the abundance of CMP of the whole microbial community are shown in Supplemental Fig. 11. The gut microbiome metabolic pathways of arginine and proline metabolism (arginine, hydroxyproline), histidine metabolism (histamine), BAs metabolism (cholic acid, glycine, taurine), alanine, aspartate and glutamate metabolism (fumaric acid), pyruvate metabolism (pyruvate), and purine metabolism (thymidine) are among the ones most correlated with paired serum metabolite concentrations (Supplemental Fig. 11A). The lumen metabolite correlation result was highly similar to that of serum, with additional relations to interconnected pathways such as the β-alanine metabolism (pantothenic acid) and polyamines (spermidine) (Supplemental Fig. 11B). Interestingly, many of these metabolic pathways were also significantly altered by tacrolimus or antibiotics treatment. The individual bacterial species that contain the genetic potentials correlating with metabolite measurements are listed in Supplemental Table 5. For example, species containing the BA hydrolase gene (choloylglycine hydrolase, cbh) include *Acutalibacter muris*, *Bacteroides thetaiotaomicron*, *Enterobacter cloacae*, *Clostridium celerecrescens*, *Lactobacillus johnsonii*, *Akkermansia muciniphila*, suggesting their potential involvement. This analysis was limited to genes with annotated KEGG BRITE metabolic reactions, which comprised 44.7% and 43.3% of all serum and fecal metabolites, respectively. Some important metabolites, such as tryptophan indole pathway compound IPA, which were significant in our metabolome analyses, could not be included. Based on *in silico* modeling and previous knowledge of microbe-derived metabolites [59], the major metabolic pathways attributed to the tacrolimus metabotype are likely microbiome-dependent.

### Antibiotics and tacrolimus, alone and synergistically, rapidly modulate lymph node (LN) and intestinal immune compartments

**Fig. 8:**
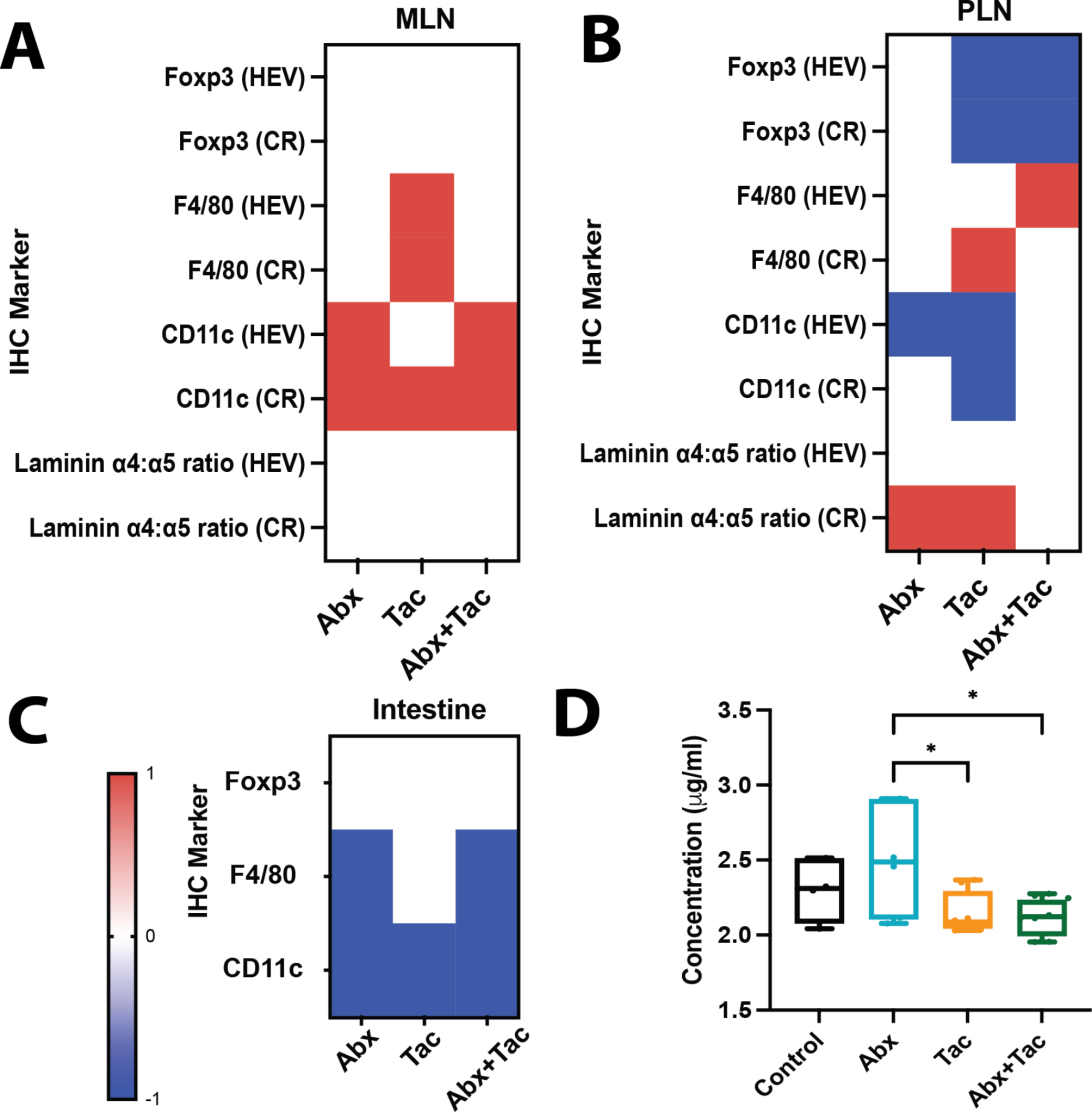
Changes in the distribution of lymphocytes and myeloid cells and LN structure due to abx and tacrolimus. Mice given abx (6 days) with or without 2 days of tacrolimus. Representative qualitative heatmaps of IHC marker changes relative to untreated control (red = increased; blue = decreased; white = unchanged) (**a**) mLN, (**b**) pLN, and (**c**) intestine. Graphs of individual IHC values for each marker and tissue type listed in Supplementary Fig. 12. (d) In vivo gut permeability assay performed using FITC-Dextran. Mice were gavaged with FITC-Dextran and serum samples were collected post 4 h of administration. 3 mice/ group, at least 2 mLN, pLN, and sections of intestine at duodenal-jejunal junction/mouse, 3 sections/staining panel. Ordinary one-way ANOVA with Tukey’s multiple comparisons test. Representative of 2 repeated experiments. * p < 0.05; ** p < 0.01, *** p < 0.001, **** p < 0.0001. *Abbreviations*: LN: lymph node; mLN: mesenteric LN; pLN: peripheral LN; IHC: immunohistochemistry

To comprehensively understand the drug-induced immunosuppressive environment, a triangulation of metabolome, microbiome, and immune characteristics is important. We further assessed immune system structure by flow cytometry of important leukocyte subsets in mesenteric LN (mLN) and peripheral LN (pLN), and by immunohistochemistry for these same subsets in LNs and intestine, and for stromal laminins in LNs. Flow cytometry showed no significant differences in the overall cellularity of CD4 + T cells, CD8 + T cells, Foxp3 + Tregs, and B220 + B cells in the mLN or pLN in any of the groups after two days of treatment (Supplemental Fig. 12). Immunohistochemistry (IHC) showed that F4/80 + macrophages (MΦ) were significantly increased in the mLN around the high endothelial venules (HEV) by tacrolimus compared to the other groups (Fig. 8A). CD11c + dendritic cells (DCs) were increased around the HEV and within the cortical ridge (CR) for all treatment groups, especially in the combined treatment group (Fig. 8B). In the pLN, Foxp3 + Tregs decreased in the CR and around the HEV in the tacrolimus treatment groups, both with and without antibiotics, but not with antibiotics alone (Fig. 8B). CD11c + DCs decreased in the pLN CR and around HEV in all treatment groups, but most significantly in the tacrolimus treatment group (Fig. 8B). In the pLN CR, laminin a4:a5 ratios were highly increased by tacrolimus-only treatment and slightly increased by antibiotics-only treatment (Fig. 8B). In the intestine, Foxp3 + Tregs were also slightly increased by tacrolimus compared with antibiotics and antibiotics with tacrolimus (Fig. 8C, Supplemental Fig. 12c). Similarly, F4/80 + MF in the intestine was increased by tacrolimus alone compared to antibiotics, both with and without tacrolimus (Fig. 8C). Assessment of intestinal barrier integrity using FITC-dextran revealed a marked increase in gut permeability following antibiotic treatment compared to tacrolimus alone or combined treatments (Fig. 8D). This suggests that antibiotic-induced alterations to the gut microbiota might disrupt the mucosal barrier, potentially increasing the risk of bacterial translocation and subsequent systemic inflammatory responses. Interestingly, while tacrolimus, when administered alone, did not significantly impact barrier function, its co-administration with antibiotics appeared to ameliorate the detrimental effects on the barrier caused by antibiotics. Alternatively, the immunosuppressive properties of tacrolimus may play a role in mitigating local inflammatory responses that can contribute to barrier disruption. Overall, our study underscores the importance of considering the combined effects of treatments on gut barrier function, especially when targeting conditions where both antibiotics and immunosuppressants are commonly prescribed. Further, tacrolimus displayed rapid anti-inflammatory properties within two days of treatment, and this effect was distinct from that of the other groups.

## Discussion

Immunosuppressive drugs, while critical in organ transplantation, are imprecise treatments that can cause unintended changes in microbial and host metabolism [4, 5]. While metabolic complications are generally accrued over time and thus progressive, this study focused on the early metabolic changes after drug treatment, aspects that have been largely uncharacterized. Our results showed significantly altered metabolism in both circulation and gut lumen within 2 days of tacrolimus treatment, widely affecting multiple metabolic classes, including a group of interconnected amino acid metabolisms, BA conjugation, glucose homeostasis, and energy production. Following a 7-day treatment course, the metabolic landscape was again altered to a different state, reflecting more pronounced shifts in the gut microbiome and extensive perturbation in amino acid metabolites. These alterations, exhibiting strong inter-correlations, underscore the incremental drug effects on the microbiome and metabolic processes. These results have significant implications in that diverted metabolism in mammalian hosts controls the alloimmune response and bioavailability of amino acids [72]. Crucial mechanisms demonstrated in this study involve increased local catabolism and production of amino acid-derived metabolites via synergistic reactions by the microbiome, intestinal epithelia, and host organs connected through circulation. Our study highlights the early dynamics of metabolic changes. These bioactive molecules, representing the specific metabotype, provide potential targets for early diagnostics and therapeutics for metabolic disorders due to immunosuppressant drug use.

Tacrolimus displayed rapid anti-inflammatory properties within two days of treatment, as shown by the increased pLN laminin a4:a5 ratios. Antibiotics are broadly pro-inflammatory, signified by a decrease in intestinal Tregs and increased gut permeability. These findings reinforce the notion that antibiotics and immunosuppressants, alone and together, exert rapid and distinct pro- and anti-inflammatory perturbations of the LN and gut immune structures. Together, our results emphasize the profound and rapid impact of immunosuppressants on various facets of the immune system and critical physiological processes including the unintended alteration in metabolism and the balance of gut microbiome. It remains unclear whether the drug impact on immunity caused changes in metabolic activities and the microbiome, or if there were multiple interactions so that immunity, metabolism, and microbiome all influenced each other simultaneously.

The dosage and selection of immune suppressants and the antibiotic cocktail used in this study must be considered in data interpretation, as they are simplified compared to actual clinical use. Tacrolimus is not the sole immunosuppressive medication administered post organ transplantation. Typically, it is used in conjunction with an antimetabolite, mycophenolate mofetil, as well as glucocorticoids. Multiple anti-bacterial, -viral, and -fungal antibiotics are used at the time of transplantation and for many months post-transplantation. Our use of tacrolimus was to mimic clinical observations, without the over immunosuppression that occurs in mice receiving clinical triple immunosuppression protocols and superior to murine models based on acute, binary measures of rejection [38, 39]. The moderate tacrolimus doses reflect clinical management with formation of chronic graft lesions and alloreactivity, mirroring ineffective immune suppression that plagues human recipients, within an experimentally tractable duration. Since there is the widely variable use of multiple antibiotics in clinical settings, we employed a previously characterized regimen in mice in which multiple broad-spectrum oral antibiotics were used to deplete the endogenous gut microbiota [73]. This antibiotic cocktail, though different from what is used clinically, was effective in removing the “barricade” effect of gut microbiota in murine models, allowing the subsequently introduction of treatments such as immune suppressants or FMT of whole stool or selected strains to study their effects. By using antibiotics prior to immunosuppression, we aimed to “normalize” the gut microbiome baseline in which the effects of the immunosuppressive agent could be distinctly studied without the potential compounding effects of ongoing antibiotic administration. We previously used this model to demonstrate pro-inflammatory and anti-inflammatory effects of microbiota FMT [38]. To increase the model relevance, we included an experimental group that sequentially combined both antibiotics and tacrolimus treatment. Future experiments are warrant where both treatments will be administered concurrently to better replicate the clinical context and validate our findings. Nonetheless, it will be important to incorporate additional parameters and refine our existing murine model to further elevate its clinical relevance.

This study strongly supports further in-depth characterization of the distinct metabotype and microbiome metabolic profile, an aggregation of selected metabolic and microbial features that reflect functional alterations [74] due to an immunosuppressed environment. Though dysbiosis has long been known to significantly contribute to metabolic disorders [75], the underlying metabolic shift in response to immunosuppression remains unclear. Our study provides evidence for the indispensable role of the gut microbiome in systemically affecting host metabolism. Altered primary BA conjugation and deconjugation strongly supported the involvement of gut microbiota after tacrolimus treatment to regulate the gut-liver BA cycle. Changes in the hepatic metabolism of BAs also affect the gut microbiota, which in turn regulates immune function and gut inflammation [76]. Changes in the availability of nutrients by the drug and/or changes in the gut microbiome that affect host digestion and absorption may also lead to an altered need for certain amino acids. Future mechanistic investigations will be crucial to unravel the multifaceted impacts of tacrolimus. This includes characterizing its direct impact on the gut microbiota and subsequent production of bioactive molecules, as well as discerning whether tacrolimus first influences host metabolome, which in turn modulates microbial activities. Such depth of knowledge will pave the way for devising novel therapeutic strategies targeting these interactions, optimizing patient outcomes and potentially mitigating adverse effects associated with prolonged immunosuppression.

We designed this study to understand the disruption to the metabolome repertoire of bioactive molecules that reflect the interactions among gut microbiota, immunosuppressant drugs, and immune responses under immune suppressant drug treatment. There are multiple limitations to this study. The sample size was small thus limiting the statistical power analyses. This study nevertheless indicated significant and intricate metabolic changes in an immune suppressed environment. We intentionally focused on the early metabolic changes, characterizing them at 2 and 7 days. This emphasis on early changes is important to identify immediate shifts in metabolic activities, which might have profound implications even before overt clinical symptoms manifest. A comprehensive investigation, both well-powered with temporal monitoring, will be essential to delineate metabolic signatures that correspond to short-, intermediate- and long-term effects of the drug. We noted that we used only one immunosuppressant. While validated in our murine model for both relevance and feasibility, further comparison and/or combining with other immunosuppressive drugs will provide additional knowledge about metabolic changes. A mechanistic understanding of the metabolic changes in an immunosuppressed environment has the potential to redefine the management of transplantation patients concerning the long-term adverse effects of immunosuppression to achieve optimized health outcomes.

## Methods and materials

### Study approval

All procedures involving mice were performed in accordance with the guidelines and regulations set by the Office of Animal Welfare Assurance of the University of Maryland School of Medicine under the approved IACUC protocol nos. 1,518,004 and 0121001.

### Mice experiments

Female C57BL/6 mice between 8 and 14 weeks of age were purchased from The Jackson Laboratory (Bar Harbor, ME, USA) and maintained at the University of Maryland School of Medicine Veterinary Resources breeding colony. We only used female mice for the current set of experiments where we worked with smaller n-values, to ensure a high degree of homogeneity within our study groups. All procedures involving mice were performed in accordance with the guidelines and regulations set by the Office of Animal Welfare Assurance of the University of Maryland School of Medicine. Mice were fed antibiotics (kanamycin, gentamicin, colistin, metronidazole, and vancomycin) *ad libitum* in drinking water on days 7 to -1. Antibiotics were USP grade or pharmaceutical secondary standard (all from MilliporeSigma): kanamycin sulfate (0.4 mg/ml), gentamicin sulfate (0.035 mg/ml), colistin sulfate (850 U/ml), metronidazole (0.215 mg/ml), and vancomycin hydrochloride (0.045 mg/ml) were dissolved in vivarium drinking water. Mice received daily immunosuppression of tacrolimus (3 mg/kg/d subcutaneously) on days 0, 1 [77, 78]. Tacrolimus (USP grade, MilliporeSigma) was reconstituted in DMSO (USP grade, MilliporeSigma) at 20 mg/ml and diluted with absolute ethanol (USP grade, Decon Labs, King of Prussia, PA) to 1.5 mg/ml. DMSO/ethanol stock was diluted 1:5 in sterile phosphate buffered saline (PBS) for subcutaneous injection and injected at 10 µl/g (3 mg/kg/day) [77, 78]. All mice were cohoused and handled together during arrival in the animal facility and for antibiotic and immunosuppressant administration so that the various treatment groups were all exposed to each other. On day 2, the mice were euthanized by CO_2_ narcosis. Intralumenal stool samples were collected for metagenomic and metabolomic analyses. At the time of euthanasia, we utilized cardiac puncture for blood collection. We acknowledge the potential effects of first-pass hepatic metabolism on general circulation. Over extended periods, however, there should be a normalization of systemic metabolites throughout the body, unless there are acute changes in the intestine that we did not observe within the experimental timeframe. Portal blood provides a more direct measure of metabolites absorbed from the intestine before they undergo hepatic metabolism. However, in mice, portal blood can only be reliably obtained at the time of euthanasia, and the obtainable volumes are exceedingly low, making its routine collection challenging. Mesenteric and peripheral (axillary, inguinal, and brachial) LNs, as well as the small intestine, were harvested for immunohistochemistry. The mLN, pLN, and spleen samples were collected for flow cytometry analyses. Mouse experiments were performed according to ARRIVE guidelines (https://arriveguidelines.org).

### Flow cytometry

LNs were disaggregated and passed through 70-µm nylon mesh screens (Thermo Fisher Scientific, Waltham, MA) to produce single-cell suspensions. Cell suspensions were stained for 30 min at 4 °C with antibodies against surface molecules (Supplemental Table 6) and washed 2 times with FACS buffer [PBS with 0.5% w/v bovine serum albumin]. Cells were permeabilized using Foxp3/Transcription Factor Staining Buffer Set (eBioscience, San Diego, CA) according to manufacturer’s protocol, washed with FACS buffer, and subsequently stained at 4 °C with antibodies for intracellular molecules. Samples were analyzed with an LSR Fortessa Cell Analyzer (BD Biosciences), and data were analyzed using FlowJo software version 10.6 (BD Biosciences). Single color controls (cells stained with single surface marker antibody) and unstained controls were used for flow channel compensation. Representative gating strategy provided in Supplemental Figure S13.

### Immunohistochemistry

Mesenteric and peripheral LN and segments of the intestine between the duodenum and jejunum were separately excised and immediately submerged in OCT compound (Sakura Finetek, Torrance, CA, USA) or fixed using paraformaldehyde. Cryosections (5 μm) were cut in triplicate using a Microm HM 550 cryostat (ThermoFisher Scientific) and fixed in cold 1:1 acetone:methanol for 5 min, washed in PBS, or left unfixed for fluorescent microscopy. Sections were rehydrated in PBS and blocked with 2.5% donkey serum and 2.5% goat serum in PBS. The sections were then stained at room temperature with primary antibodies (diluted 1:20 − 1:200 in PBS), blocked with 10% secondary antibody host serum, incubated with secondary antibodies (diluted 1:50 − 1:400 in PBS) for 30 min, fixed with 4% paraformaldehyde in PBS for 5 min, quenched with 1% glycerol in PBS for 5 min, and mounted with Prolong Gold Antifade Mountant with or without DAPI (Thermo Fisher Scientific). Images were acquired using a Nikon Accu-Scope EXC-500 (Nikon, Tokyo, Japan) and analyzed using Volocity software (PerkinElmer, Waltham, MA). The antibodies used are listed in Supplemental Table 6. Three mice/group, 3–4 mesenteric LN or peripheral LN or pieces of intestine, 3–6 sections/tissue sample, and 10–15 fields/tissue sample were analyzed. The mean fluorescence intensity (MFI) was calculated within demarcated high endothelial venules (HEV) and cortical ridge (CR) regions of the mLN and pLN as well as of whole intestinal images. The percent area was calculated by dividing the sum area of demarcated regions with marker fluorescence greater than a given threshold by the total area analyzed. Treatment groups were compared using quantification of MFI multiplied by percent area to express both the area and intensity of cell and stromal fiber markers. Qualitative heat maps were generated (GraphPad prism) to express changes in IHC marker expression level relative to control using 1 to represent “increased,” 0 to represent “unchanged,” and − 1 to represent “decreased.”

### In vivo intestinal permeability assay

Intestinal permeability was assessed as described previously [79]. Briefly, mice were gavaged with FITC-dextran (Catalog# 46,944, MW:4000; Sigma, St. Louis, MO, USA) at a dose of 60 mg/100 g body weight at a concentration of 120 mg/mL. Four hours later, after euthanasia, blood was collected via cardiac puncture and allowed to clot at room temperature for 2 h in the dark. Tubes containing blood were centrifuged at 10,000 x g for 10 min at 4 °C, and the supernatant serum was collected. FITC-dextran serum concentration (µg/ml) was measured in duplicate using a black, flat-bottom, 96-well plate (Greiner Bio-one, Frickenhausen, Germany) on a FlexStation 3 Microplate Reader (Molecular Devices, San Jose, CA) at an excitation wavelength of 490 nm and emission wavelength of 530 nm.

### Statistics

Datasets were analyzed using GraphPad Prism 9.3.1 (San Diego, CA, USA) with statistical significance defined as *P* < 0.05. For comparisons of fluorescent markers (including laminin α4:α5 ratios), serum markers, and inflammation scores, Tukey’s multiple comparison tests of one-way ANOVA were used to test for significance.

### Stool specimen collection, DNA extraction, and metagenomic sequencing

The jejunum and colon tissues of the mice were dissected according to their gastrointestinal anatomical features. Intraluminal stool contents were collected from dissected tissues and stored immediately in DNA/RNA Shields (Zymo Research, Irvine, CA, USA) at -80 °C to stabilize and protect the integrity of nucleic acids and minimize the need for immediate processing or freezing of specimens. DNA extraction was described previously [39, 80]. In brief, 0.15–0.25 g of fecal samples were extracted using the Quick-DNA Fecal/Soil Microbe kit (Zymo Research, Irvine, CA, USA). Negative extraction controls were included to ensure that no exogenous DNA contaminated the samples. Metagenomic sequencing libraries were constructed using the Nextera XT Flex Kit (Illumina), according to the manufacturer’s recommendations. Libraries were then pooled together in equimolar proportions and sequenced on a single Illumina NovaSeq 6000 S2 flow cell at Maryland Genomics at the University of Maryland School of Medicine.

### Gut microbiome analyses

Metagenomic sequence reads were removed using BMTagger v3.101 [81] mapping to Genome Reference Consortium Mouse Build 39 of strain C57BL/6J (GRCm39) [82]. Sequence read pairs were removed when one or both the read pairs matched the genome reference. The Illumina adapter was trimmed and quality assessment was performed using default parameters in fastp (v.0.21.0) [83]. The taxonomic composition of the microbiomes was established using Kraken2 (v.2020.12) [84] and Braken (v. 2.5.0) [85] using the comprehensive mouse gut metagenome catalog (CMGM) [43] to calculate the metagenomic taxonomic composition. Phyloseq R package (v1.38.0) [86] was used to generate the barplot and diversity index. In the context of our study, the mice gut microbiome datasets contain a broad spectrum of taxa without any dominant high-abundance species. As such, the Chao1 diversity index, an abundance-based indicator of species richness (total number of species in a sample) that is sensitive to low abundance taxonomic groups (singletons and doubletons) [87], was employed. This methodological choice ensures accurate representation and understanding of the microbial community diversity in our samples. Linear discriminant analysis (LDA) effect size (LEfSe) analysis [88] was used to identify fecal phylotypes that could explain the differences. The α value for the non-parametric factorial Kruskal-Wallis (KW) sum-rank test was set at 0.05 and the threshold for the logarithmic LDA model [89] score for discriminative features was set at 2.0. An all-against-all BLAST search was performed in the multiclass analysis. Microbial biomarkers were calculated using the limma voom function [90] in R package microbiomeMarker v1.3.3 [91]. Phylogram representing the taxonomic hierarchical structure of the identified phylotype biomarkers via pairwise comparisons between groups, graph generated using R package yingtools2 [92]. The metagenomic dataset was mapped to the protein database UniRef90 [42] to ensure the comprehensive coverage in functional annotation, and was then summarized using HUMAnN2 (Human Microbiome Project Unified Metabolic Analysis Network) (v0.11.2) [41] to efficiently and accurately determine the presence, absence, and abundance of metabolic pathways in a microbial community. Canonical Correspondence Analysis (CCA) was used for ordination analysis using the vegan package [48, 93] based on the Bray-Curtis distance. CA1 and CA2 were selected as the major components based on their eigenvalues.

### Metabolite extraction and metabolome analyses

Metabolome of intraluminal stool (luminal/local) and serum (circulating/systemic) were measured using capillary electrophoresis-mass spectrometry (CE/MS) to obtain a comprehensive quantitative survey of metabolites (Human Metabolome Technologies, Boston, MA, USA). ~10–30 mg of stool was weighed at the time of collection using a company-provided vial and archived at -80 °C at the IGS until shipped to the HMT on dry ice. QC procedures included standards, sample blanks and internal controls that were evenly spaced among the samples analyzed. Compound identification was performed using a CE/MS library of > 1,600 annotated molecules.

Selecting a proper data pretreatment method is essential in metabolomic data analyses to reduce the influence of measurement noise [94]. The normalization procedures were performed using the combination of sample normalization for general-purpose adjustment for systematic differences among samples, log base 10 transformation was applied to individual values themselves, and data scaling adjusted each variable/feature by a scaling factor computed based on the range of each variable as the dispersion of the variable. Metabolites were exhaustively annotated using known metabolic databases or the *a priori* knowledge-based approach, achieved using PubChem [44], KEGG [45], and HMDB [46] annotation frameworks that leverage cataloged chemical compounds, known metabolic characterization, and functional hierarchy (i.e., reaction, modules, pathways). The sparse PLS-DA (sPLS-DA) algorithm implemented using mixOmics (vers. 6.18.1) was employed to analyze the large dimensional datasets that have more variables (metabolites) than samples (p > > n) to produce robust and easy-to-interpret models [47]. The “sparseness” of the model was adjusted by the number of components in the model and the number of variables within each component based on the classification error rate with respect to the number of selected variables. Tuning was performed one component at a time, and the optimal number of variables to select was calculated. The volcano plot combines results from FC analysis to show significantly increased metabolites after 7-day tacrolimus treatment. A metabolite is shown if FC is > 2 and the p-value is < 0.05 based on 2-sample t-tests. Original metabolite measurements without normalization were used in the FC analysis. To evaluate the discriminative capacity of a target metabolite in distinguishing between experimental groups, ROC curves were constructed. These curves plotted the sensitivity against 1-specificity across various threshold settings, thereby offering a comprehensive overview of the metabolite’s classification performance over the complete range of possible decision boundaries. The Area Under the Curve (AUC) derived from the ROC curve serves as a metric quantifying the overall ability of the metabolite to correctly classify the experimental conditions. The optimal threshold was identified as the point on the ROC curve nearest to the top-left corner (denoted by a red dot), as this position represents the trade-off value between sensitivity and specificity. For the corresponding box plots depicting the distribution of metabolic measurements across experimental groups, the p-values, calculated using 2-sample t-tests, are displayed atop the boxplots. Correlation network of metabolome was performed using Debiased Sparse Partial Correlation (DSPC) network [51] implemented in MetaboAnalyst 5.0 [95, 96]. Nodes denote taxonomic groups or metabolites; edges represent association measures. Default cutoff value was used for degree filter and betweenness. Correlation significance value < 0.01 used. Sparse partial least squares (or projection to Latent Space, PLS) was used to integrate paired datasets of the same mouse in the same latent space to demonstrate their level of agreement [52, 53]. The metabolic phenotype in the tacrolimus or antibiotic groups produced more “homogeneous” sample projections, as depicted by the short average arrow length between the paired datasets. In microbiome analyses, the HMP Unified Metabolic Analysis Network and Uniref90 database were used to stratify functional profiles according to contributing species. These microbial features were annotated using the KEGG Enzyme Nomenclature (EC number system) [97] to characterize the microbiome metabolic potentials (CMP), or the set of metabolic reactions that each microbial taxon is predicted to be capable of performing. Metabolite set enrichment analysis (MSEA) was performed for the metabolites annotated in a specific functional pathway [98]. MIMOSA2 (Model-based Integration of Metabolite Observations and Species Abundances) was used to relate variation in the microbiome metabolic potentials to paired metabolite measurement [71]. The significance of the correlation between the total community-level CMP and actual metabolite measurements across all samples was calculated using a rank-based estimation. A significant correlation indicated that the metabolic potential of a microbial community is significantly predictive of metabolic levels. The same analyses were also performed to correlate the CMP of individual microbial taxa with metabolite measurements across all samples.

### Electronic supplementary material

Below is the link to the electronic supplementary material.

Supplementary Material 1

Supplementary Material 2

Supplementary Material 3

Supplementary Material 4

Supplementary Material 5

Supplementary Material 6

Supplementary Material 7

## Data Availability

The authors confirm that the data supporting the findings of this study are available within the article and indicated supplementary materials. The data that support the findings of this study are openly available; metagenome sequences were submitted to GenBank under BioProject PRJNA809764 (https://www.ncbi.nlm.nih.gov/bioproject/PRJNA809764) with the SRA study ID SRP361281. The SRA accession numbers for each sample and their biosample ID are included in Supplemental Table 1. The R codes, including each step and parameters, were deposited in github at https://github.com/igsbma/omics_paper23. Supplemental data for this article can be accessed online at https://figshare.com/account/home#/projects/173364.
