# Supplementary material for: Rapid intestinal and systemic metabolic reprogramming in an immunosuppressed environment
Source: BMC Microbiol. 2023 Dec 9;23:394. doi: 10.1186/s12866-023-03141-z (PMC10709923; doi:10.1186/s12866-023-03141-z)
Supplement: Supplementary file 1 — Supplementary Material 1 [file 12866_2023_3141_MOESM1_ESM.docx]

**Supplemental Table 6: List of primary and secondary antibodies used in this study.**

| **Target Molecule** | **Clone** | **Catalog No.** | **Dilution Factor** |
| --- | --- | --- | --- |
| ERTR7 | Polyclonal | Santa Cruz; SC-73355 | 1:100 |
| B220 | RA3-6B2 | eBioSc; 17-0452-82 | 1:200 |
| CD4 | GK1.5 | Biolegend; 100401 | 1:400 |
| CD8 | 53-6.7 | Biolegend; 100701 | 1:400 |
| CD11b | M1/70 | eBioSc; 17-0112-82 | 1:200 |
| CD11b | M1/70 | BD; 553312 | 1:200 |
| CD11c | HL3 | BD; 550283; 553801 | 1:200 |
| CD11c | N-418 | Biolegend; 117324 | 1:200 |
| F4/80 | BM8 | eBioSc; 11-4801-81 | 1:200 |
| F4/80 | BM8 | Biolegend; 123108 | 1:200 |
| Foxp3 | FJK-16s | Invitrogen; 12-5773-82 | 1:200 |
| Foxp3 | PCH101 | eBioSc; 14-4776-82 | 1:200 |
| Laminin α4 | 775830 | R&D; MAB3837 | 1:200 |
| Laminin α5 | Polyclonal | Novus Biol; NBP1-18714 | 1:100 |
| anti-rabbit IgG AF488 | Polyclonal | Jackson ImmunoResearch; 711-545-152 | 1:800 |
| anti-rabbit DL405 | Polyclonal | Jackson ImmunoResearch; 711-476-152 | 1:800 |
| anti-rabbit IgG AF647 | Polyclonal | Jackson ImmunoResearch; 711-606-152 | 1:800 |
| anti-mouse IgG AF647 | Polyclonal | Jackson ImmunoResearchl 715-605-151 | 1:800 |
| anti-rat IgG AF594 | Polyclonal | Jackson ImmunoResearch; 112-586-143 | 1:800 |
| anti-rabbit AF488 | Polyclonal | Jackson ImmunoResearch; 111-545-003 | 1:800 |
| anti-rabbit IgG AF594 | Polyclonal | Jackson ImmunoResearch; 111-585-003 | 1:800 |
