# Supplementary material for: Rapid intestinal and systemic metabolic reprogramming in an immunosuppressed environment
Source: BMC Microbiol. 2023 Dec 9;23:394. doi: 10.1186/s12866-023-03141-z (PMC10709923; doi:10.1186/s12866-023-03141-z)
Supplement: Supplementary file 4 — Supplementary Material 4 [file 12866_2023_3141_MOESM4_ESM.docx]

**Supplemental Figures**

**Supplemental Figure 1.** Comparison of relative abundance of bacterial groups in gut microbiota of different experiment groups for **A**) Firmicutes, **B**) *Lachnospiraceae*, **C**) *Oscillospiraceae*, **D**) Ruminococcaceae that belong to Firmicutes, and **E**) Bacteroidota. Cumulative relative abundance of bacteria groups shown. Phyloseq R package (v1.38.0) (McMurdie and Holmes, 2013) to generate the barplot.

**Supplemental Figure 2.** Tacrolimus effect through comparison of no treatment control versus tacrolimus group for **A**) relative abundance box plot and **B**) logarithmic linear discriminant analysis (LDA) effect size (LEfSe) (Segata et al., 2011) of the identified phylotype biomarkers; and comparison between antibiotics with or without tacrolimus for **C**) relative abundance and **D**) LEfSe of the identified phylotype biomarkers. Alpha threshold value for pairwise non-parametric Kruskal-Wallis test was 0.05 and threshold for the logarithmic LDA model score (Fisher, 1936) for discriminative features was 2.0. All-against-all comparison in multi-class analysis performed. Phylotype biomarkers calculated using limma voom function (Law et al., 2014) in R package microbiomeMarker v1.3.3 (Cao et al., 2022).

**Supplemental Figure 3.** Phylogram representing taxonomic hierarchical structure of the identified phylotype biomarkers via pairwise comparisons between **A**) antibiotics versus no treatment control, **B**) antibiotics versus tacrolimus, **C**) antibiotics with versus without tacrolimus, and **D**) no treatment control versus tacrolimus group. LEfSe employed (Segata et al., 2011) to generate the Phylogram. Alpha threshold value for the pairwise non-parametric Kruskal-Wallis test was 0.05 and threshold for the logarithmic LDA model score (Fisher, 1936) for discriminative features was 2.0. All-against-all comparison in multi-class analysis performed. Phylogram generated using R package yingtools2 (Taur, 2023).

**Supplemental Figure 4.** Metabolites contributing to the separation of each treatment group in sPLS-DA in **A**) luminal metabolome and **B**) serum metabolome. Bar length indicates loading weights of each component; contributing variables ranked from the least important (top) to the most important (bottom).

**Supplemental Figure 5.** Hierarchical clustering heatmap of metabolites of each treatment groups in **A**) lumen and **B**) serum. Top 50 features shown. Ward linkage to cluster both samples and metabolites based on Euclidean distance. Color bar indicates the scaled z-score of each feature.

**Supplemental Figure 6.** Volcano plot combines results from fold change (FC) analysis to show significantly increased metabolites after antibiotic treatment in **A**) lumen and **B**) serum. Metabolites shown if FC is >2 and p value <0.05 based on 2-sample t-tests. Original metabolite measurement without normalization was used in FC analysis.

**Supplemental Figure 7.** Microbial biomarker analysis between 2-day and 7-day tacrolimus treatment. Phylotype biomarkers (**A**) ranked by effect size, and (**B**) taxonomic hierarchical structure in cladogram. The threshold value for the pairwise non-parametric Kruskal-Wallis test was 0.05 and the threshold for the logarithmic LDA model score for discriminative features was 2.0.

**Supplemental Figure 8.** Correlation network of **A**) gut microbiota at genus level, **B**) luminal metabolome; and **C**) serum metabolome. Debiased Sparse Partial Correlation (DSPC) network (Basu et al., 2017). Nodes are taxonomic groups or metabolites; edges represent association measures. Default cutoff value used for degree filter and betweenness. Correlation significance value <0.01 d. Both negative (blue) and positive (red) correlation coefficients included. **D**) Cluster Image Map of the Pearson correlation coefficients between two matched datasets of gut microbiota and lumen metabolome. Hierarchical clustering applied on the rows and columns of the similarity matrix simultaneously. The color represents the values of the similarity matrix when performing two dataset integration. Ward linkage clustering to cluster both samples and metabolites based on their Euclidean distance. Color bar indicates scaled z-score of each feature.

**Supplemental Figure 9.** Hydroxylated serum metabolites, which were **A**) clustered using principal component analyses and shown in **B**) hierarchical clustering heatmap. Ward linkage to cluster metabolites based on their Euclidean distance. Color bar indicates the scaled z-score of each feature.

**Supplemental Figure 10.** GCA concentration and metabolite conversion ratio plot of glycine to GCA in lumen. Conversion between GCA in serum and lumen and TCA in serum and lumen presented. Univariate ROC curve to calculate area under curve (AUC). Closest to top-left core of ROC (red dot) as the optimal cutoff value, shown in bargraph (red line). P value calculated using 2-sample t-tests. **Abbr**: CA: cholic acid; GCA: glycocholic acid; TCA: taurocholic acid.

**Supplemental Figure 11.** Top correlated metabolite measurement with the abundance of paired microbiome metabolic potentials (CMP) of the whole microbial community in **A**) serum and **B**) gut lumen. Rank-based estimation used in correlation analyses. HMP Unified Metabolic Analysis Network (v0.11.2) (Franzosa et al., 2018) and Uniref90 protein database (Kanehisa et al., 2012) used to stratify functional profiles according to contributing species. Microbial features annotated using the KEGG Enzyme Nomenclature (EC number system) (Kanehisa, 2017) to characterize CMP. MIMOSA2 (Model-based Integration of Metabolite Observations and Species Abundances) used to relate variation in the microbiome metabolic potentials to paired metabolite measurement (Noecker et al., 2016).

**Supplemental Figure 12.** Flow cytometry for CD4+ T cells, CD8+ T cells, Foxp3+ Tregs, and B220+ B cells in **(a)** mLN, pLN, and spleen of animals treated with abx (6 days) with or without 2 days of tacrolimus. IHC for **(b)** mLN F4/80+ MΦ around HEV, **(c)** mLN CD11c+ DC around HEV and CR, **(d)** pLN Foxp3+ Tregs, **(e)** pLN CD11c+ DC, **(f)** laminin 𝛼4 and 𝛼5 and ratios in pLN, **(g)** intestine Foxp3+ Tregs, and **(h)** intestine F4/80+ MΦ. 3 mice/group, at least 2 mLN, pLN, and sections of intestine at duodenal-jejunal junction/mouse, 3 sections/staining panel. Ordinary one-way ANOVA with Tukey’s multiple comparisons test. Representative of 2 repeat experiments. * p < 0.05; ** p < 0.01, *** p < 0.001, **** p < 0.0001.

**Supplemental Figure 13.** Representative gating strategy for flow cytometry analysis.

**Supplemental Tables***

*Supplemental data for this article can be accessed online at <https://figshare.com/account/home#/projects/173364>.

**Supplemental Table 1. Gut microbiome characteristics.** **A**) Statistics of metagenomic sequencing of gut microbiome and time point information. Intraluminal stool obtained from colon. **B**) Statistics of metagenomic sequencing of gut microbiome using intraluminal stool obtained from jejunum. Taxonomic assignment performed and 222 taxonomic groups **C**) at species level and **D**) at genus level characterized using the Comprehensive Mouse Gut Metagenome catalog (CMGM) (Kieser et al., 2021). **E**) Functional characterization performed using HUMAnN2 (Human Microbiome Project Unified Metabolic Analysis Network) (v0.11.2) (Franzosa et al., 2018) to determine prevalence and abundance of metabolic pathways in a microbial community.

**Supplemental Table 2.** **Metabolome of gut lumen and serum.** **A**) Luminal metabolites of the intraluminal stool and paired **B**) serum metabolites from the same mice group as indicated by mouse ID. Using KEGG BRITE hierarchical classification system (Hattori et al., 2010), the comparison between lumen and serum metabolism in **C**) KEGG functional class, **D**) pathways, **E**) modules. List of all functional modules in **F**) lumen and **G**) serum. Metabolome of intraluminal stool performed using CE/MS (capillary electrophoresis-mass spectrometry) (Kawamura et al., 2018; Matsumoto et al., 2014; Sugahara et al., 2017). Annotations of metabolites based on PubChem (Kim et al., 2021), KEGG (Hattori et al., 2010), or HMDB (Wishart et al., 2018).

**Supplemental Table 3. Metabolome comparison among different treatment groups.** Fold change (FC) analysis is to compare the absolute values of change between group means in **A**) lumen and **B**) serum. Original metabolite measurement without normalization in FC analysis. Two-sample t-test to calculate significance value. Metabolites with p value <0.05 included in the table. Pathway enrichment analysis to indicate the significantly affected pathways by treatment in **C**) lumen and **D**) serum. Enrichment analysis using predefined libraries of metabolites using metabolite set enrichment analyses (MSEA) (Xia and Wishart, 2010).

**Supplemental Table 4. Gut microbiome and metabolome after 7 days treatment of tacrolimus**. **A**) Taxonomic assignment of gut microbiota was performed to compare to gut microbiota after 2 days of tacrolimus treatment. Gut metabolome after **B**) 2 days of tacrolimus was compared to gut metabolome after **C**) 7 days of treatment. To indicate the significantly affected pathways after 7 days of tacrolimus treatment, **D**) pathway enrichment analysis was performed using metabolite set enrichment analyses (MSEA) (Xia and Wishart, 2010). Metabolites of the intraluminal stool using CE/MS (capillary electrophoresis-mass spectrometry) (Kawamura et al., 2018; Matsumoto et al., 2014; Sugahara et al., 2017).

**Supplemental Table 5: Correlation of microbiome metabolic potentials (CMP) with paired metabolite measurement.** The significance of the correlation between **A**) the total community-level CMP or **B**) individual species and actual serum metabolite measurements across all samples was calculated using rank-based estimation. Same correlation analyses were performed between **C**) the total community-level CMP or **D**) individual species and actual fecal metabolite measurements. HMP Unified Metabolic Analysis Network (v0.11.2) (Franzosa et al., 2018) and Uniref90 protein database (Kanehisa et al., 2012) were used to stratify functional profiles according to contributing species. These microbial features were annotated using the KEGG Enzyme Nomenclature (EC number system) (Kanehisa, 2017) to characterize CMP,. MIMOSA2 (Model-based Integration of Metabolite Observations and Species Abundances) was used to relate variation in the microbiome metabolic potentials to paired metabolite measurement (Noecker et al., 2016).

**Supplemental Table 6: List of primary and secondary antibodies used in this study.**

**S1**


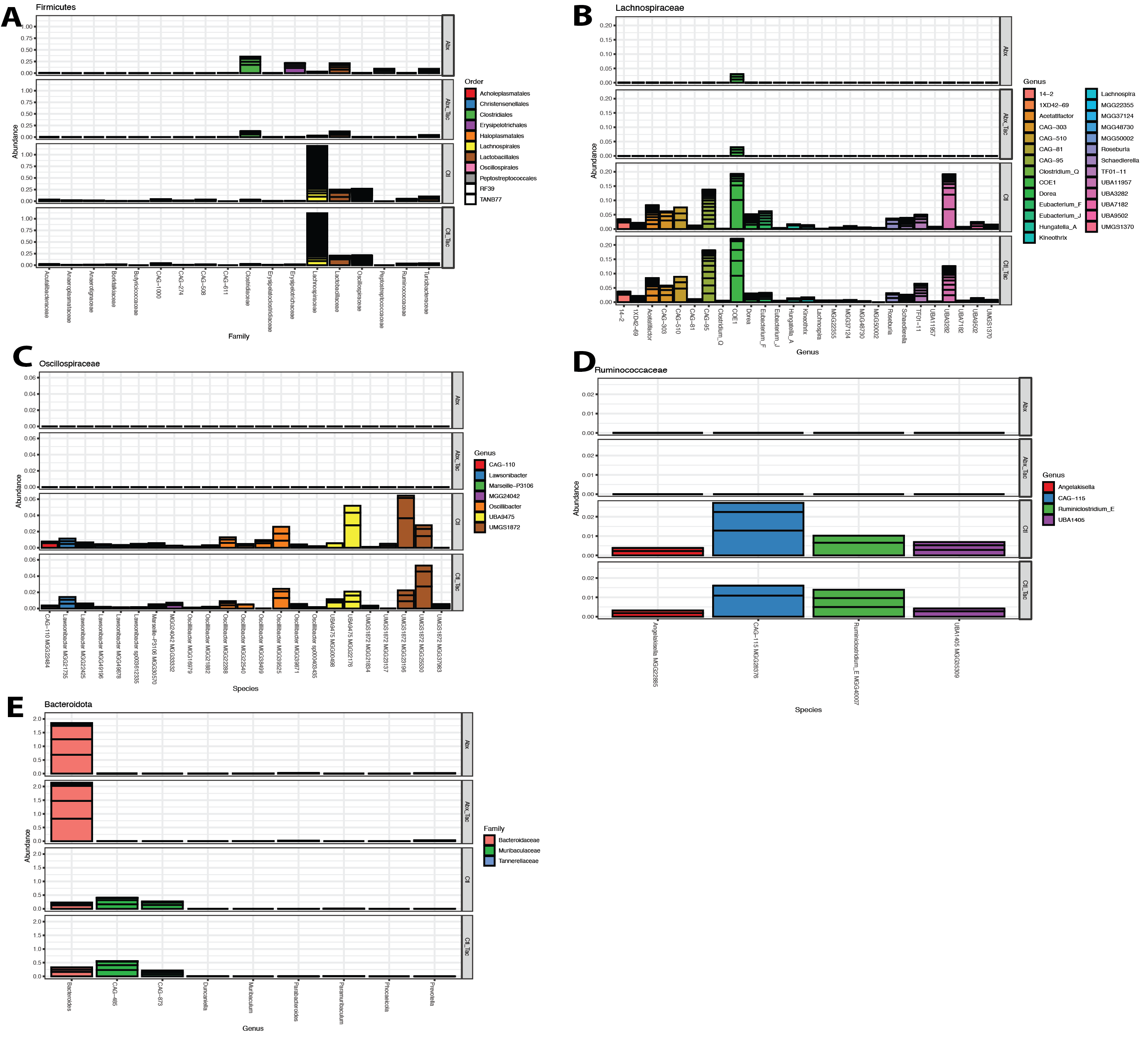


**S2.**


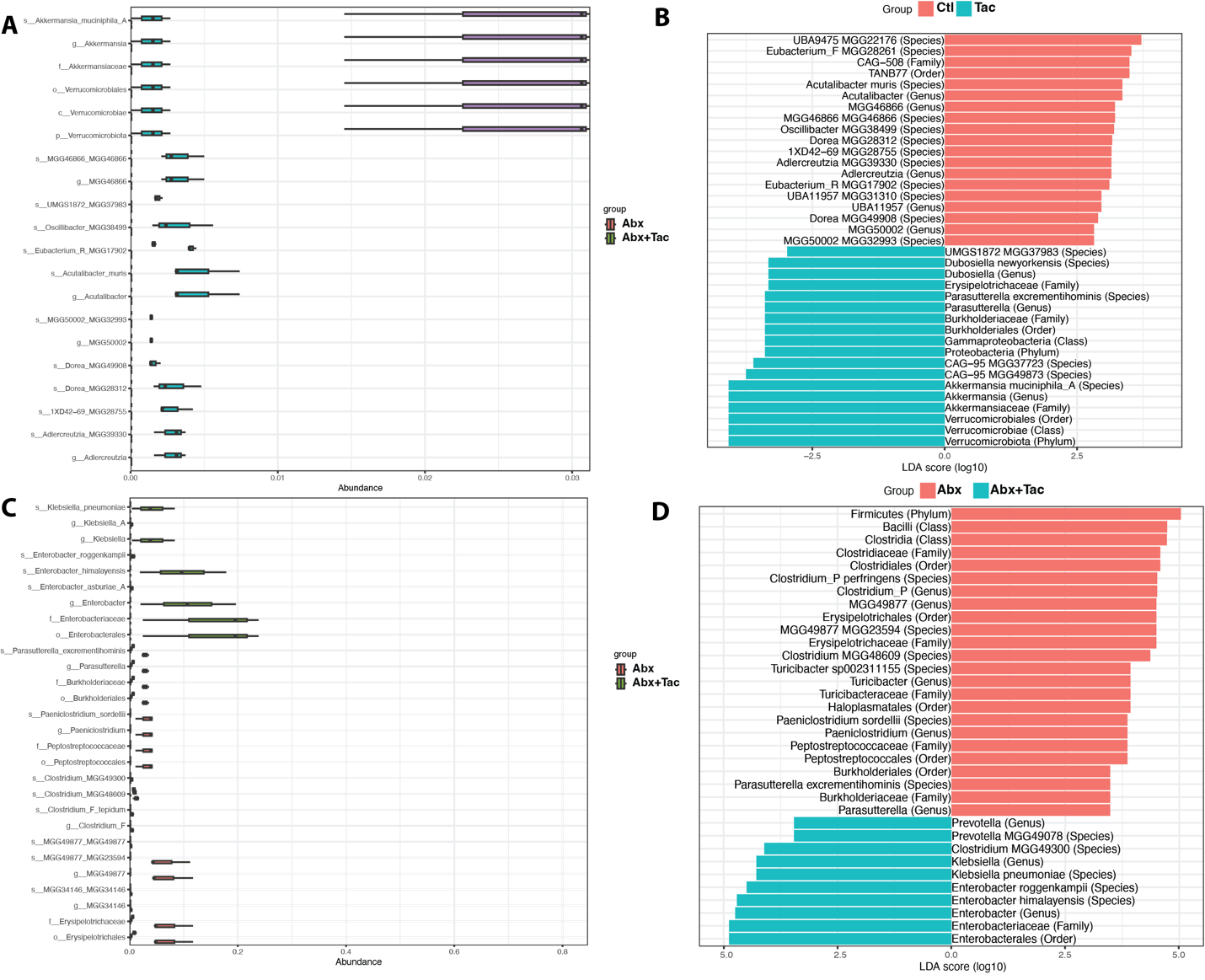


**S3**


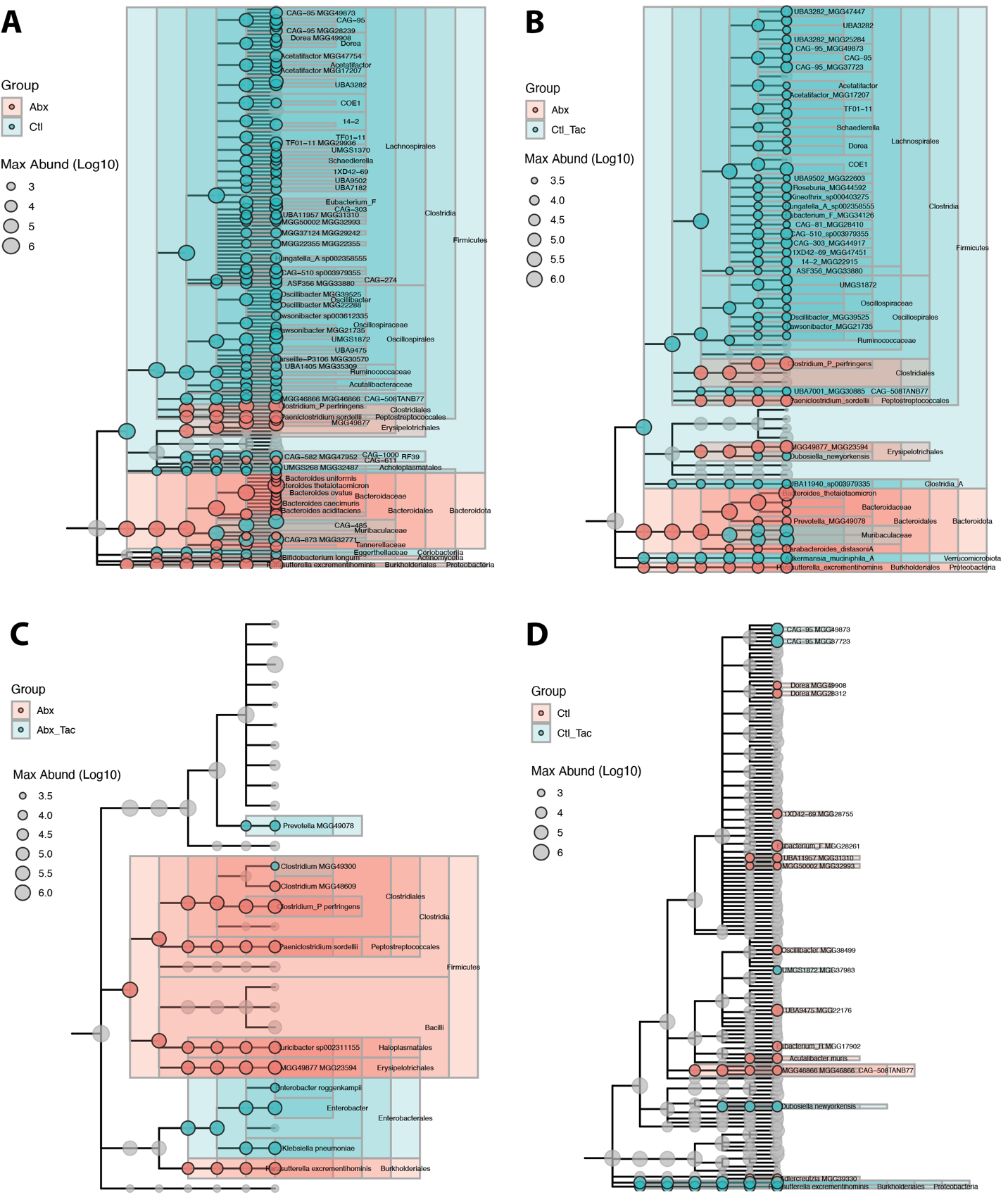


**S4.**


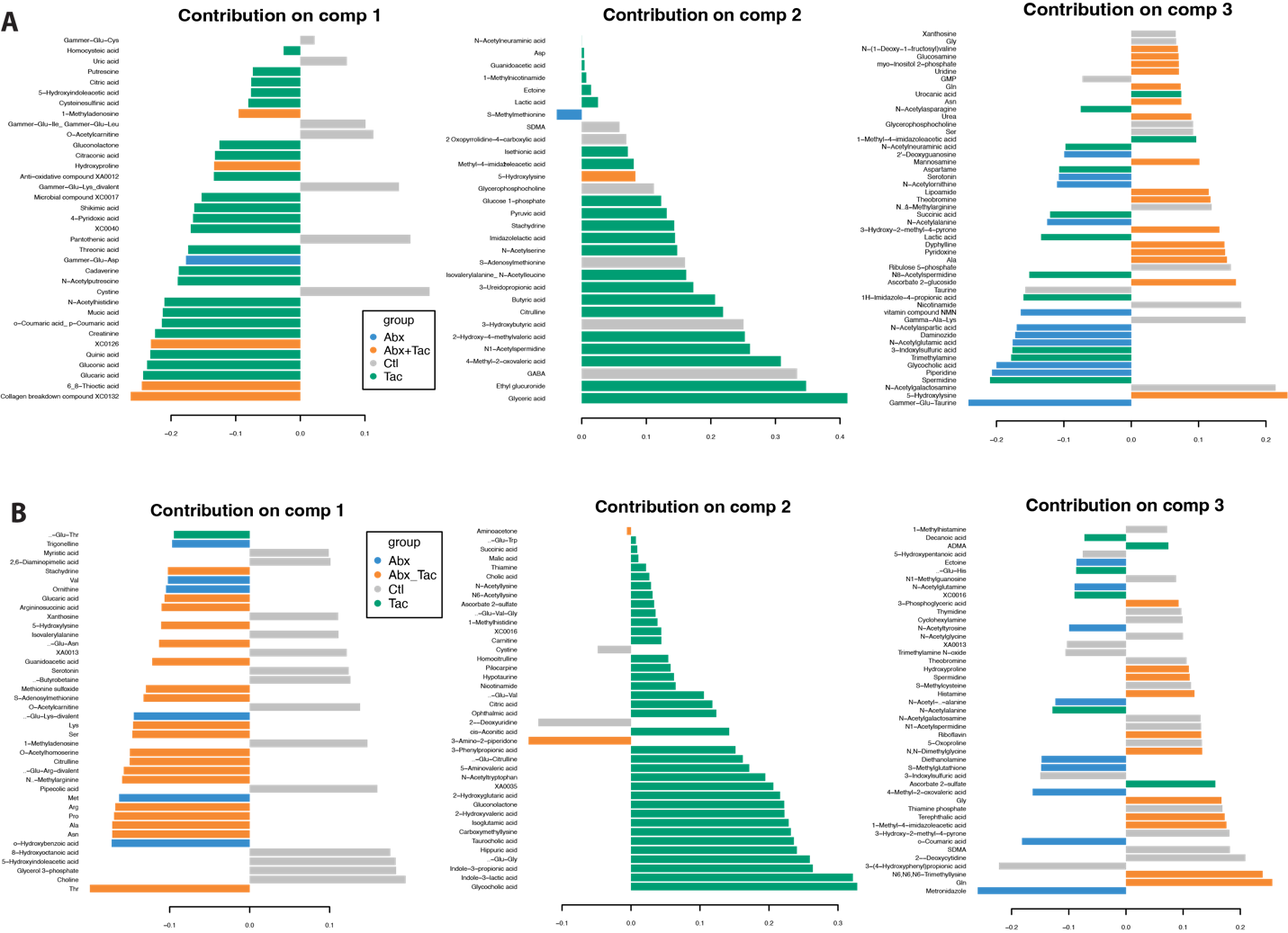


**S5.**


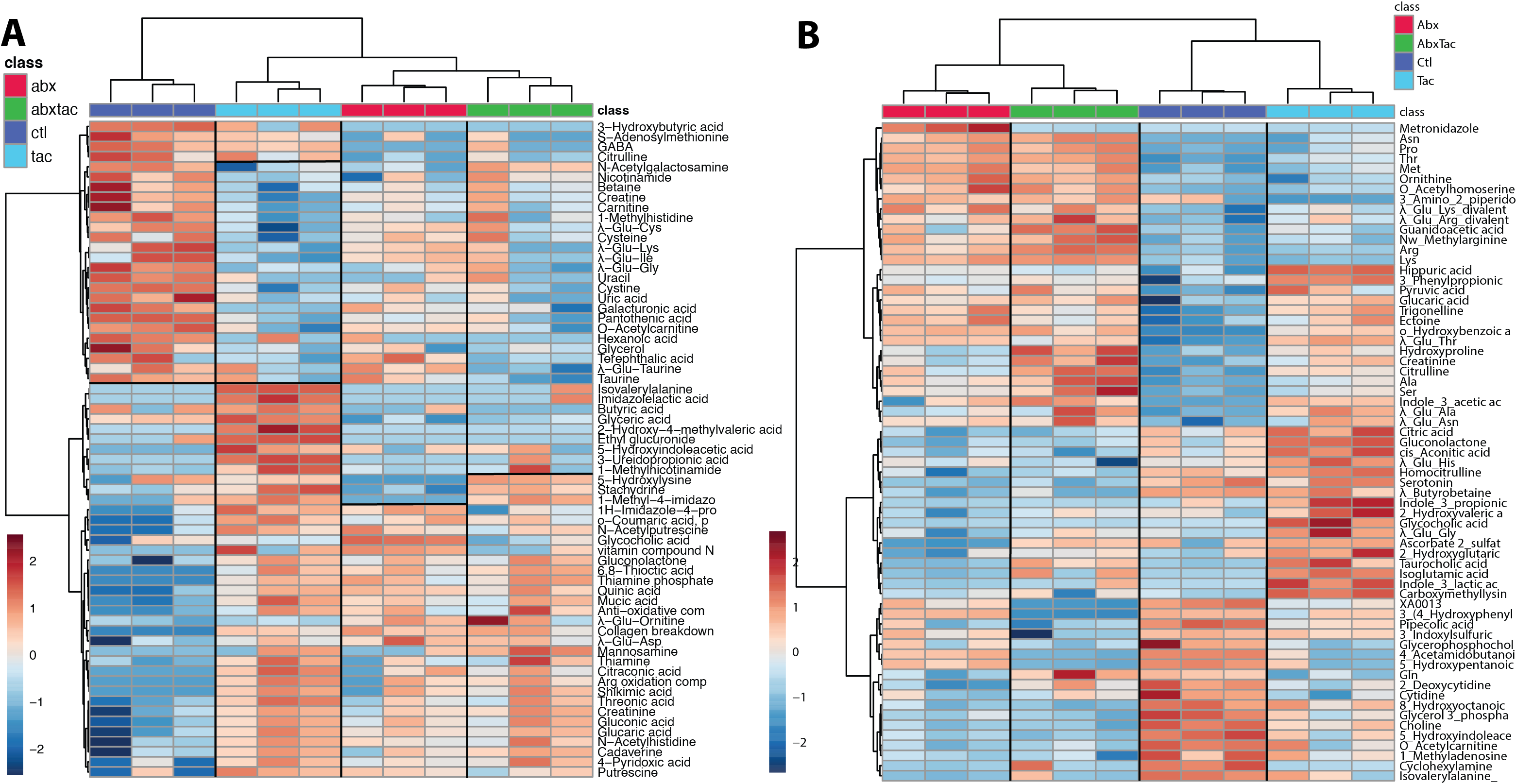


**S6.**


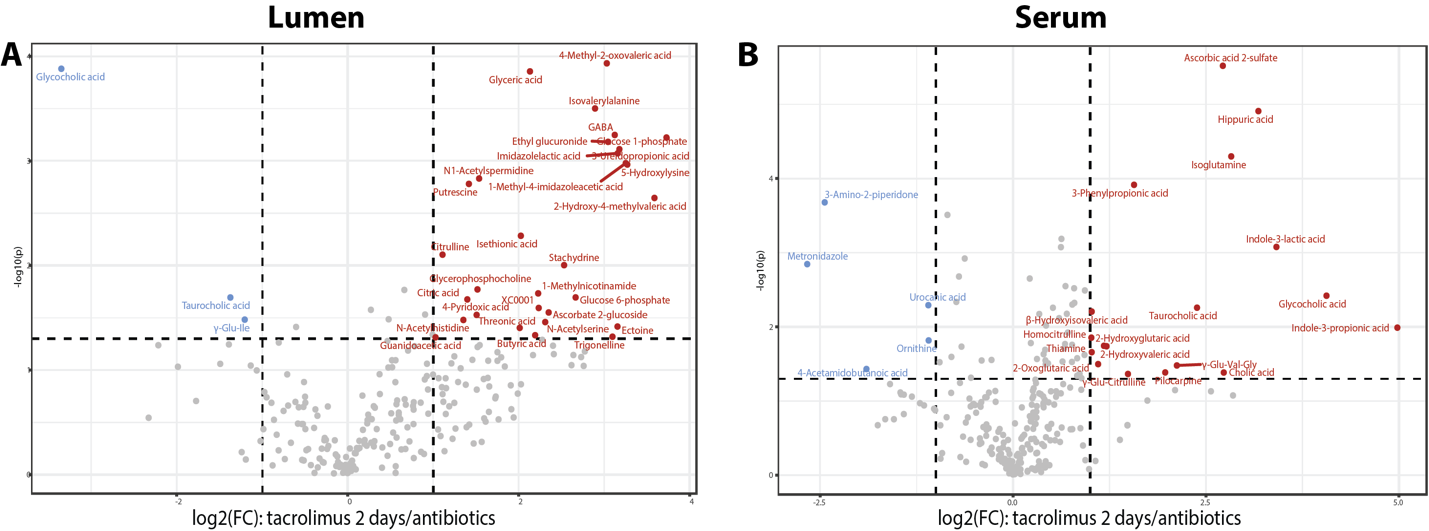


**S7.**


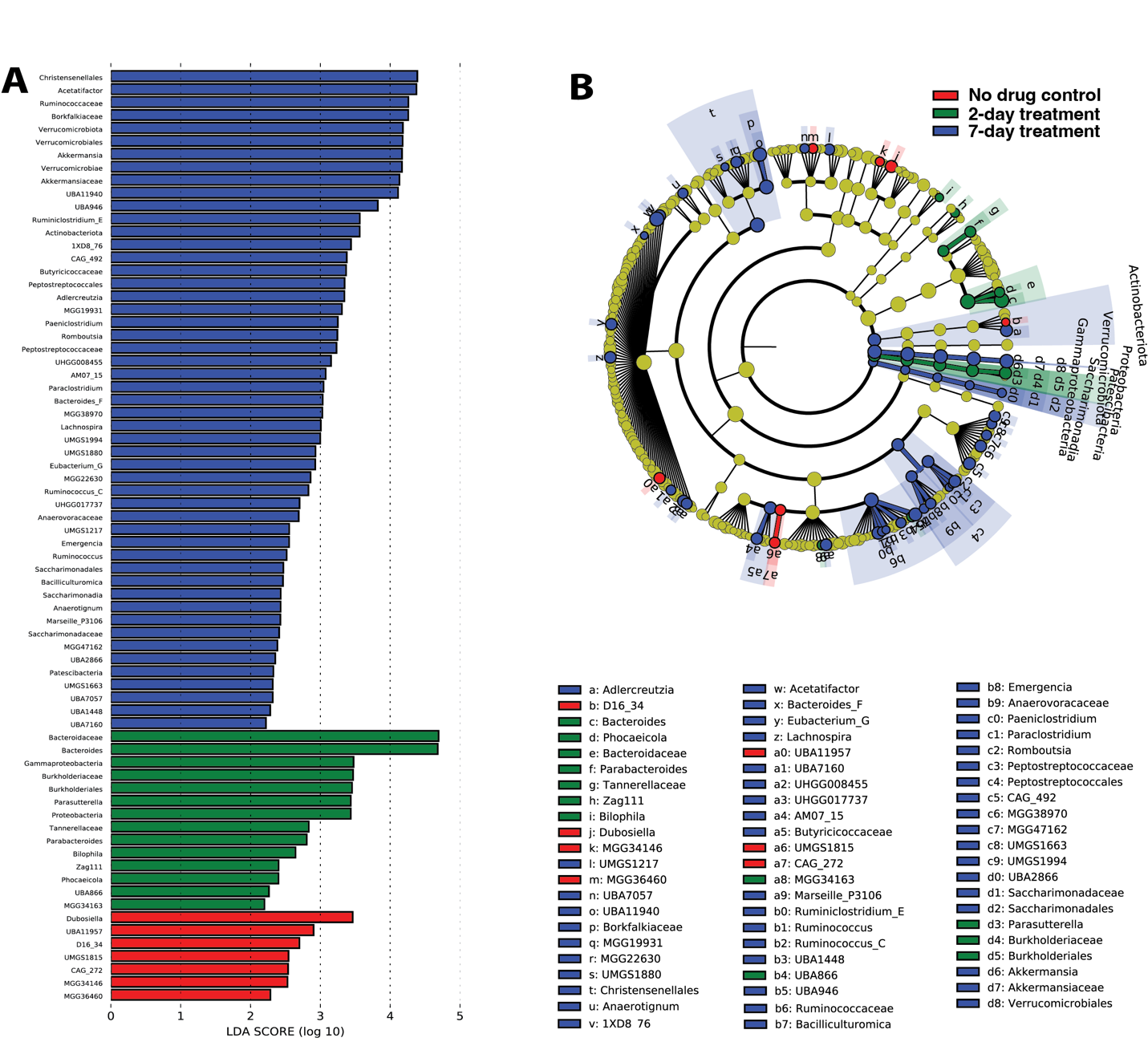


**S8.**


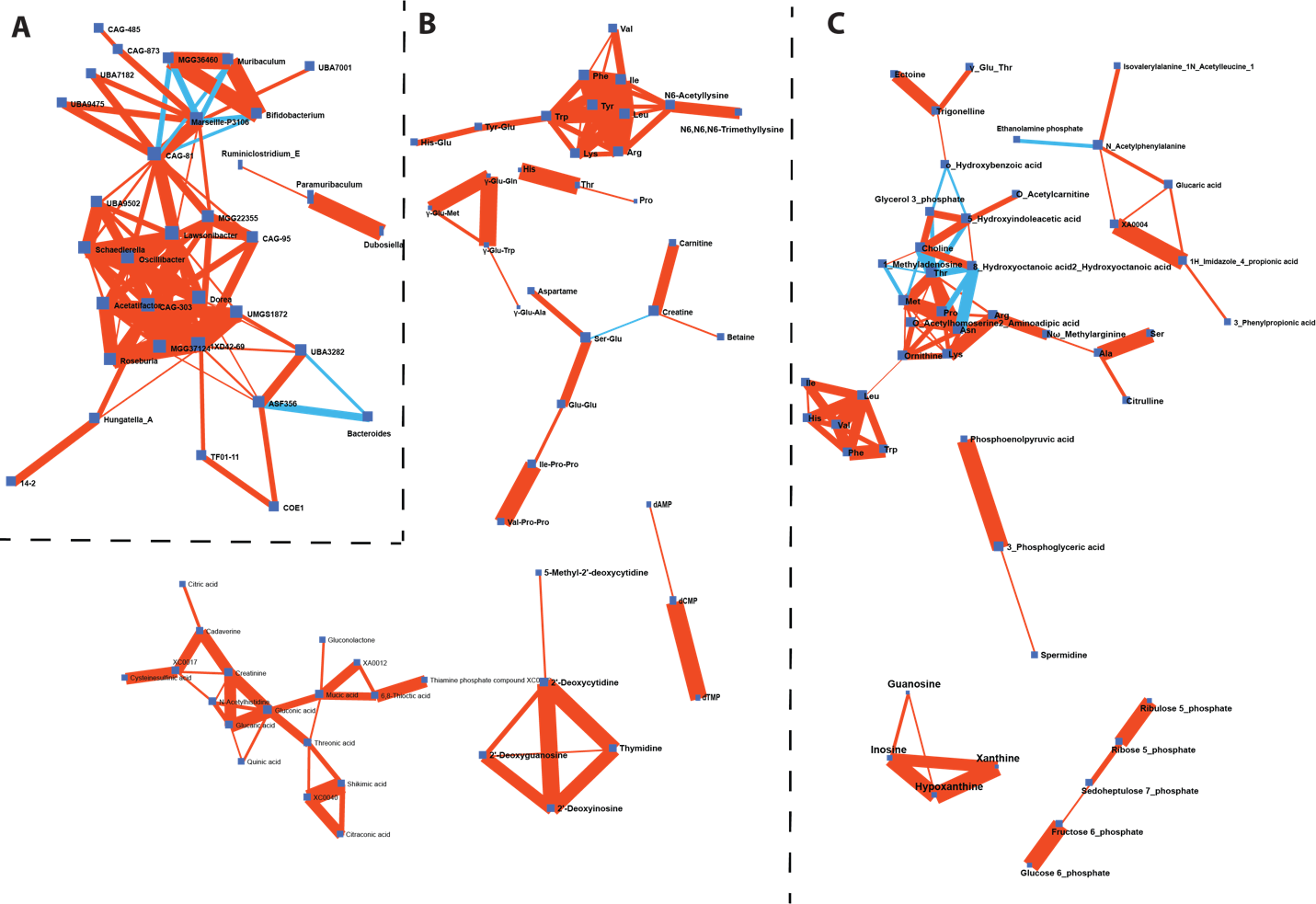


**S8 (continued)**


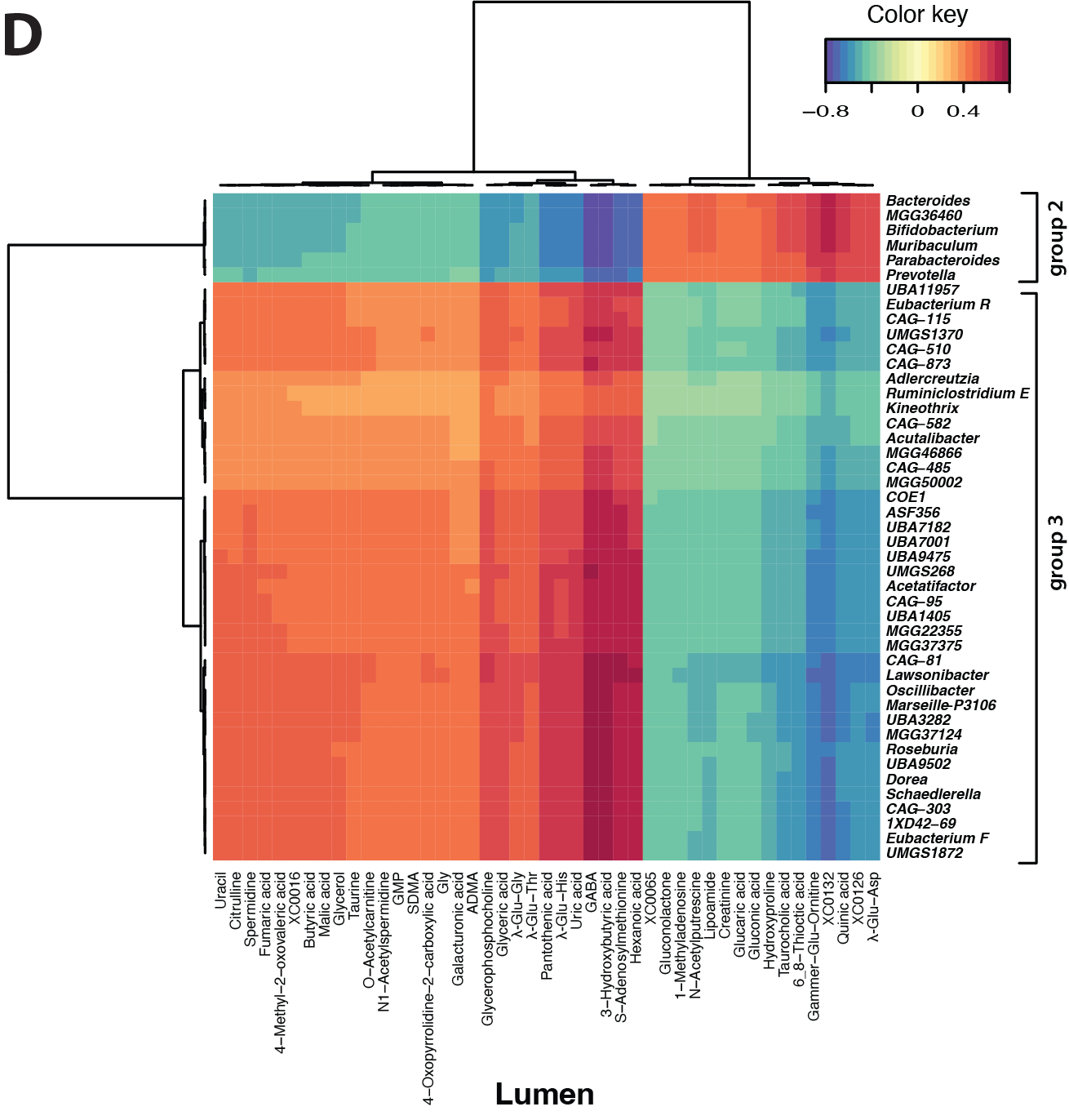


**S9.**

**
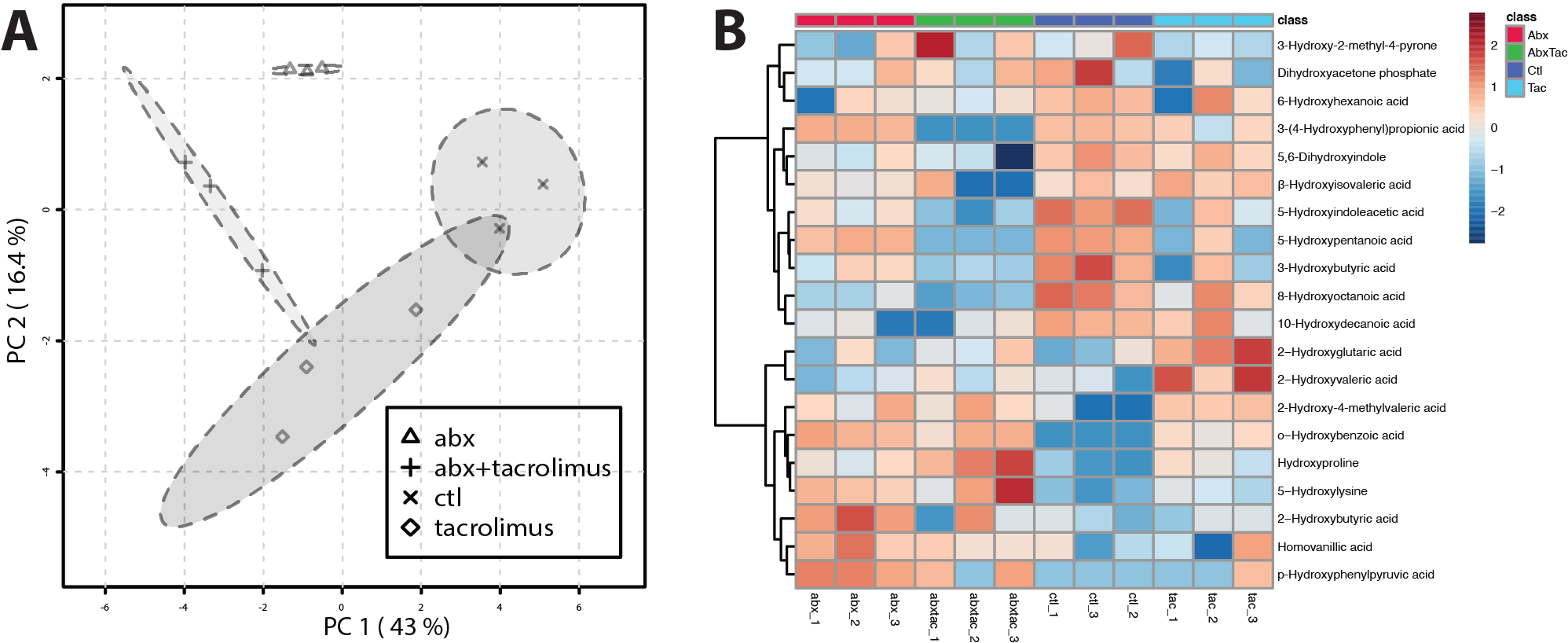
**

**S10.**


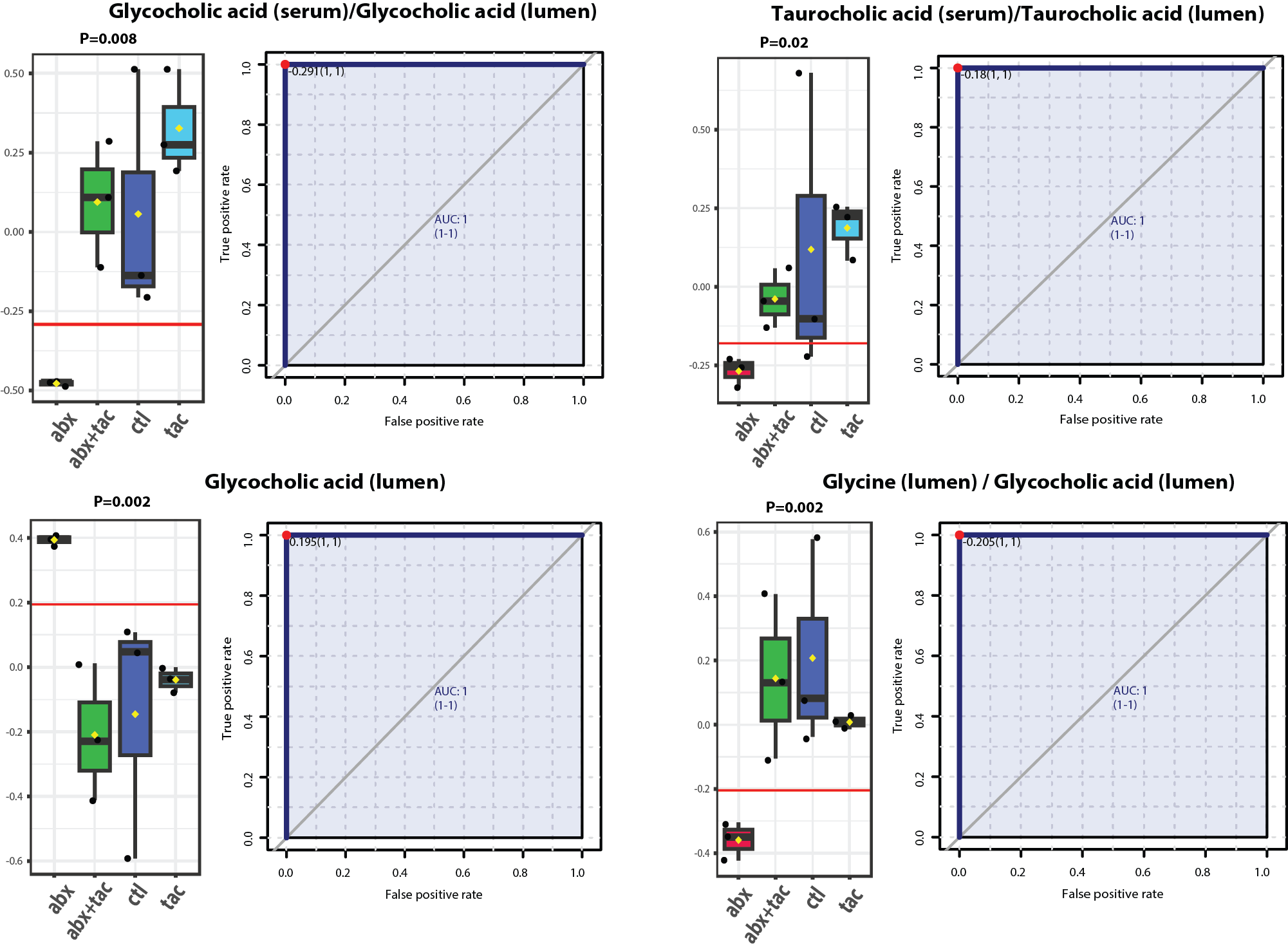


**S11.**


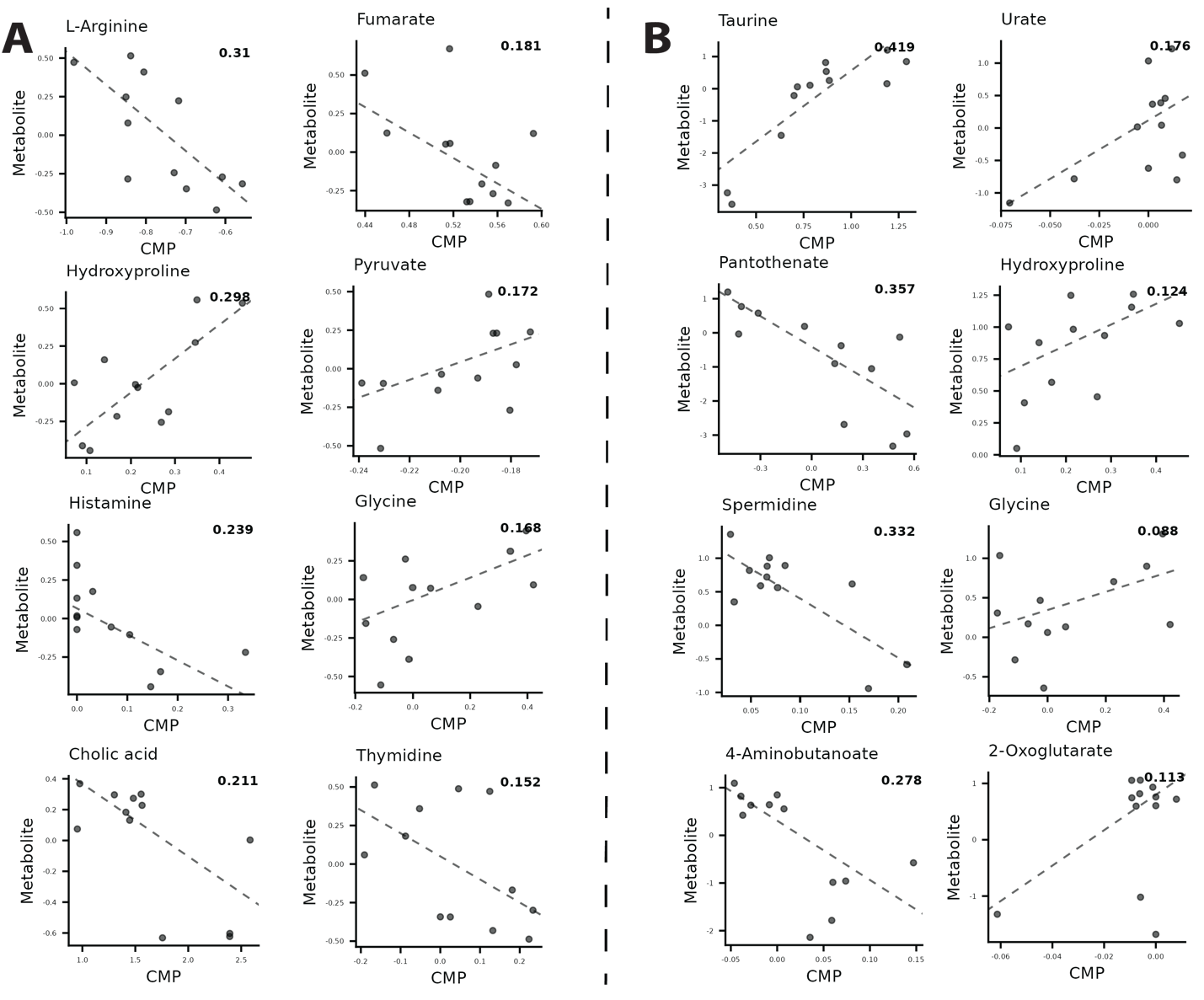


# S12.

# a.

#

#

**
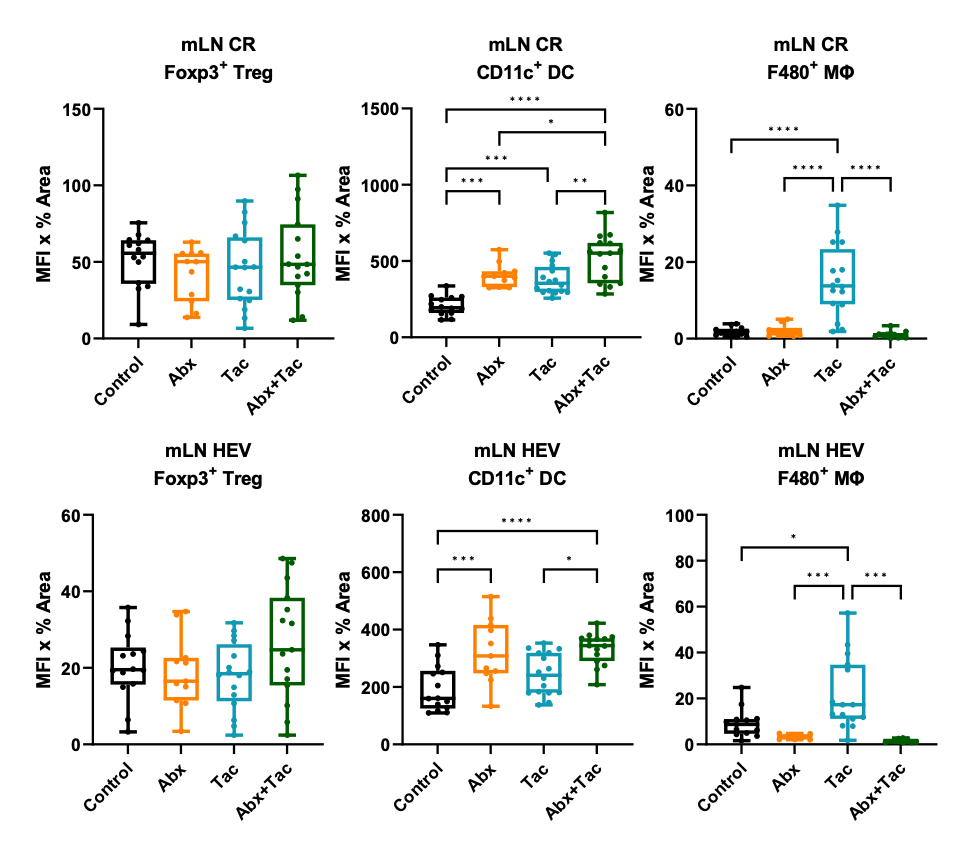
**
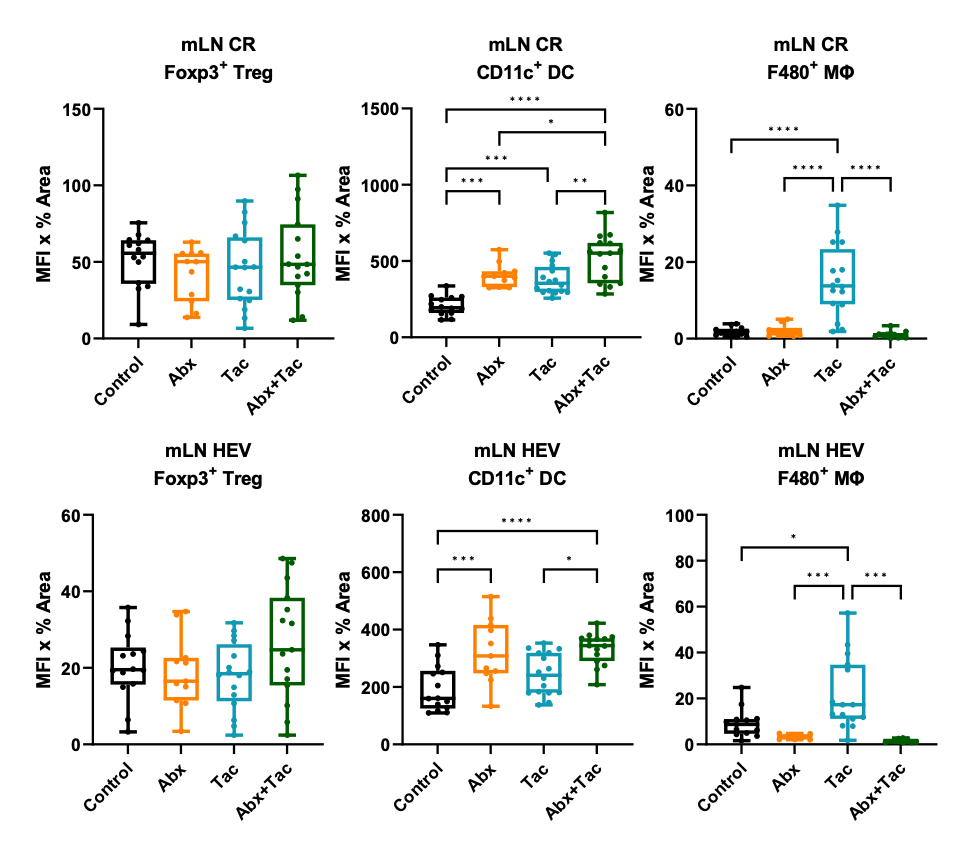
**S12 (continued).**

**
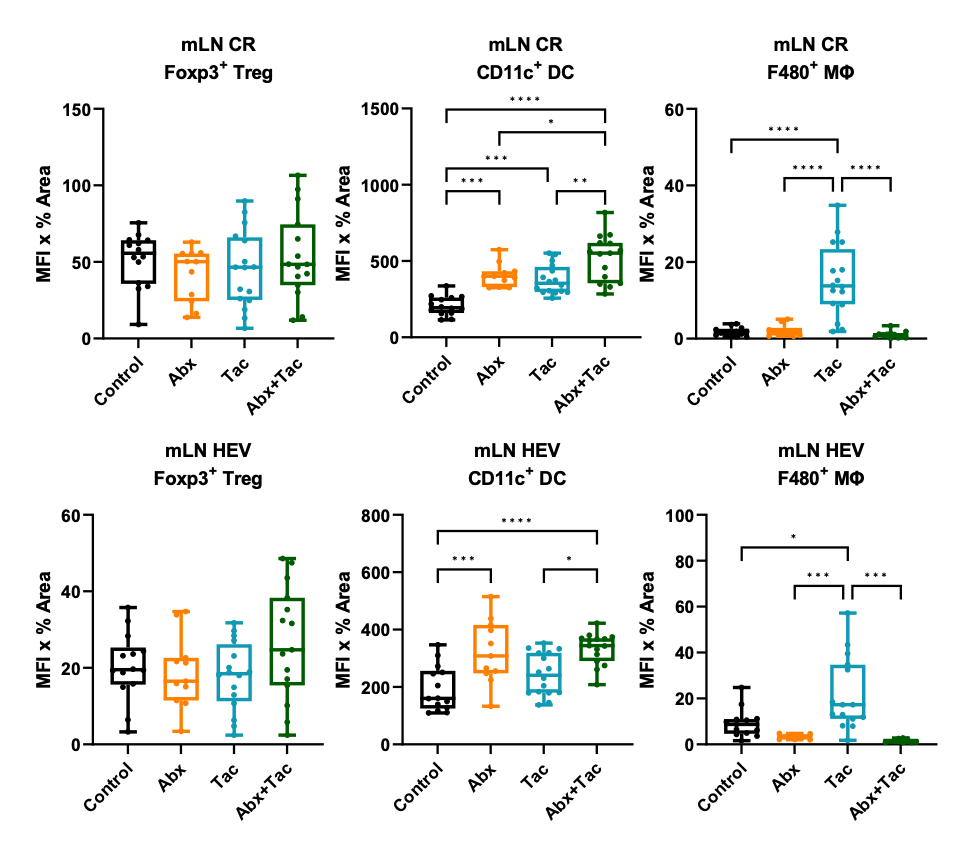
b. c.**


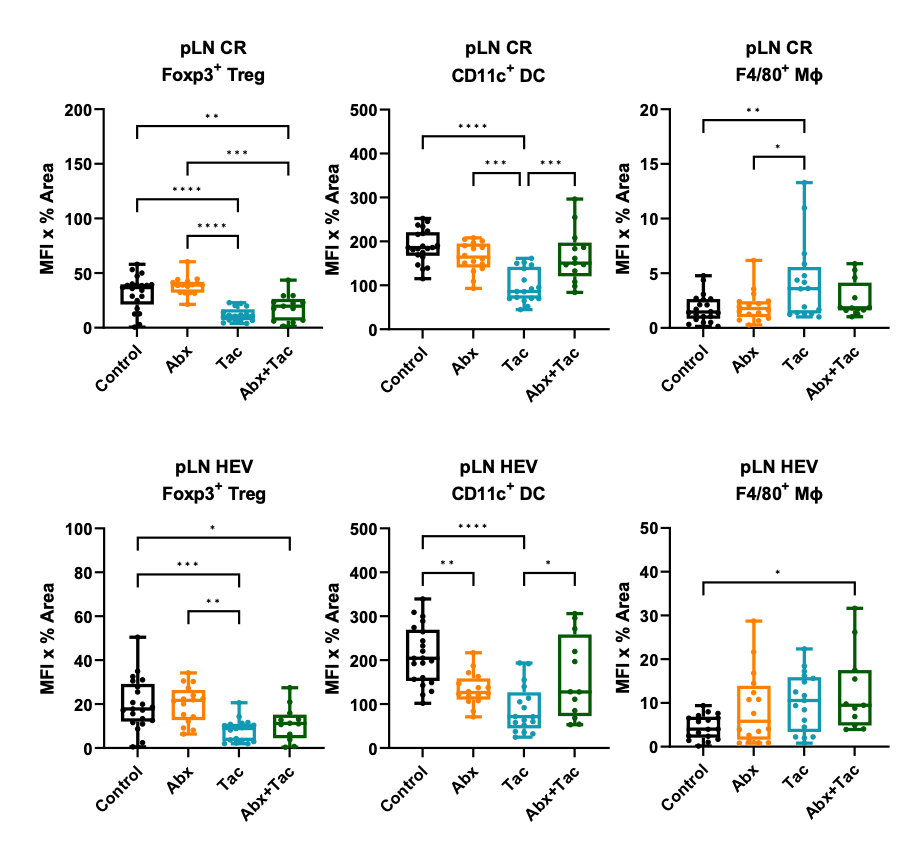


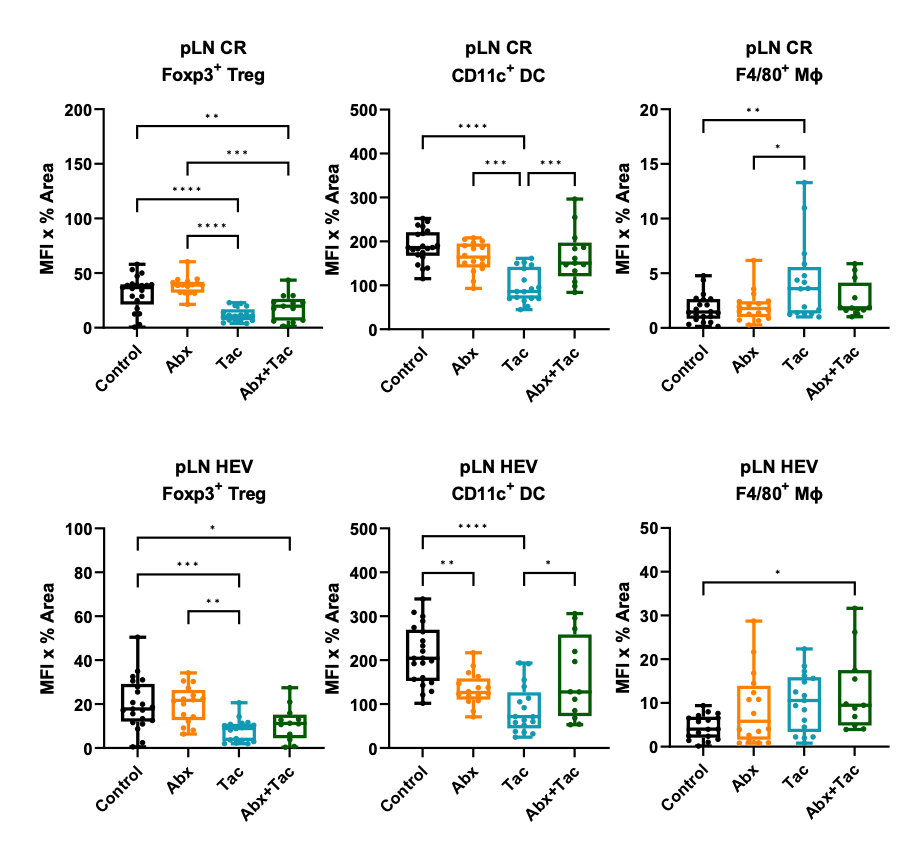


**d.**

**
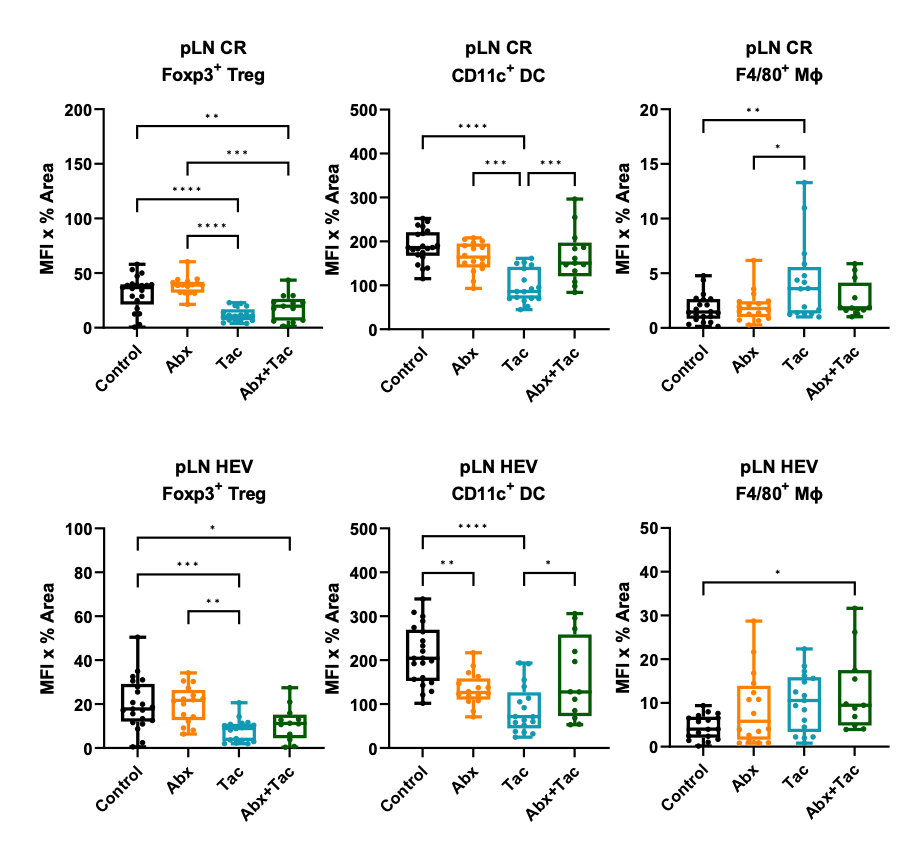

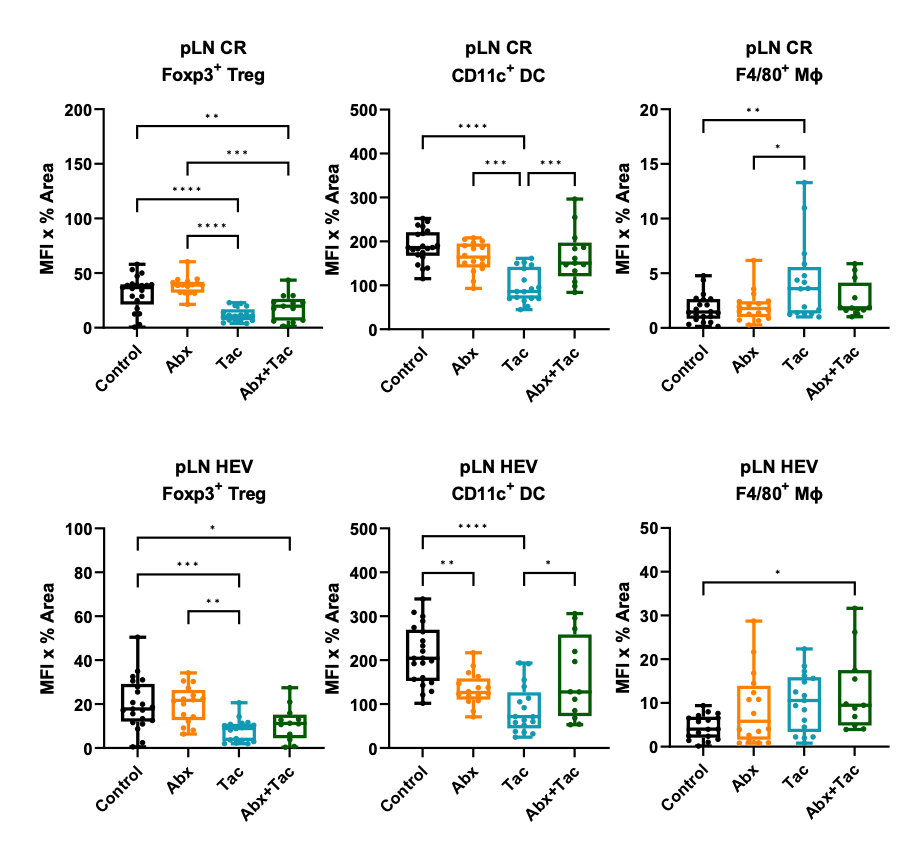
**

**e.**

**
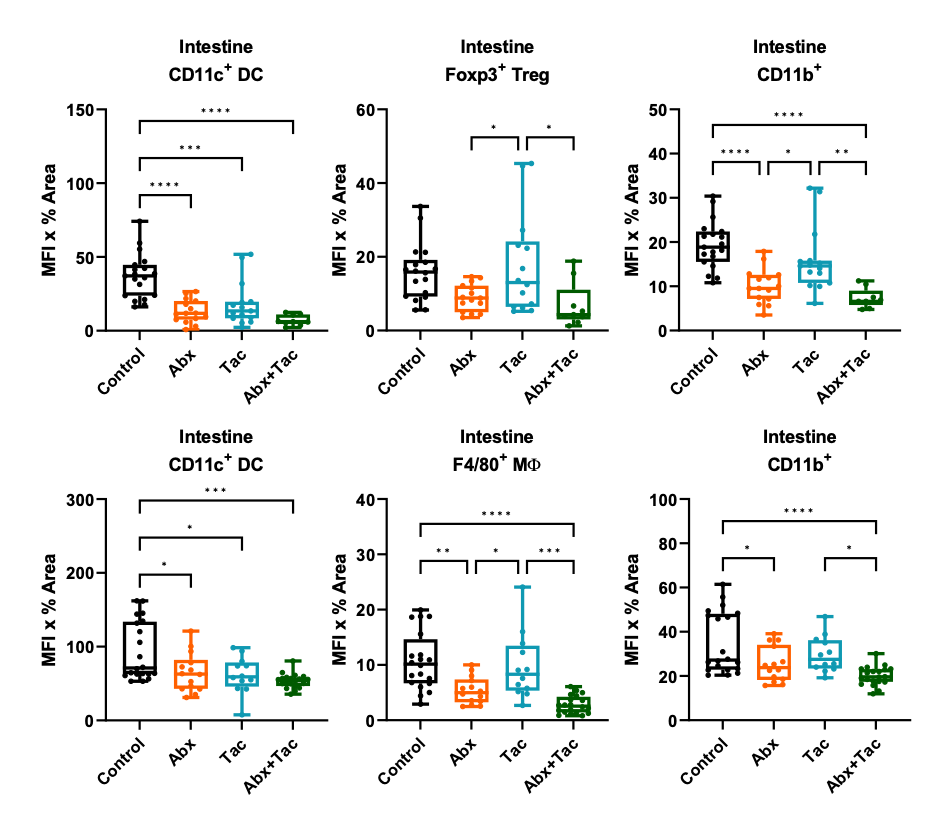

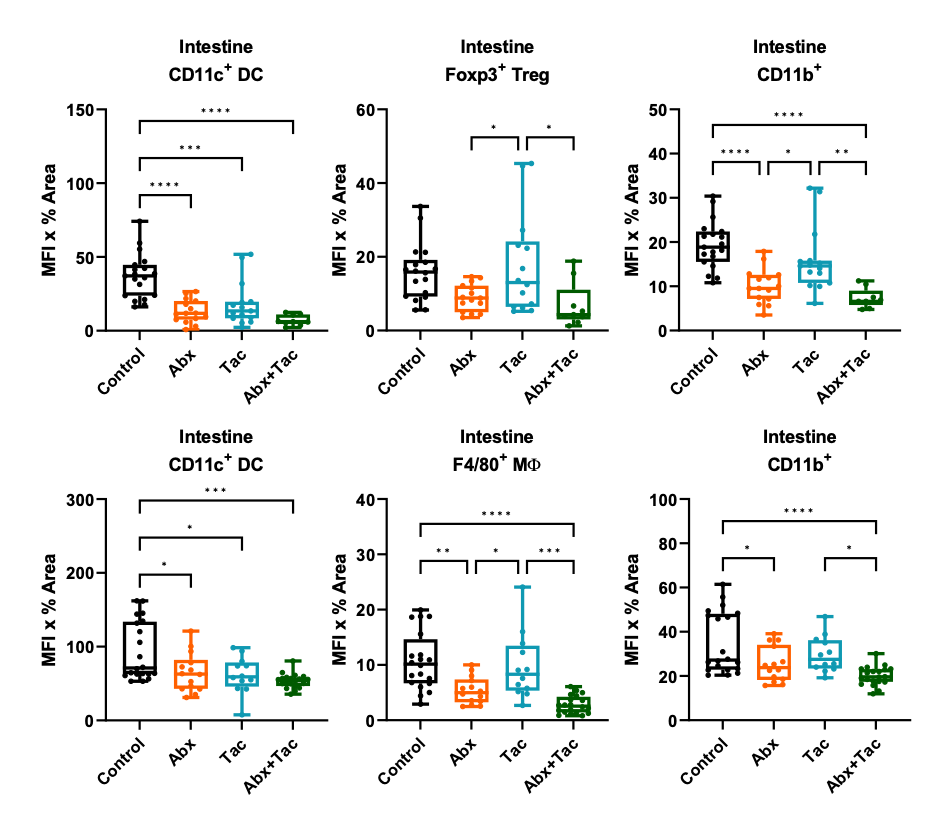

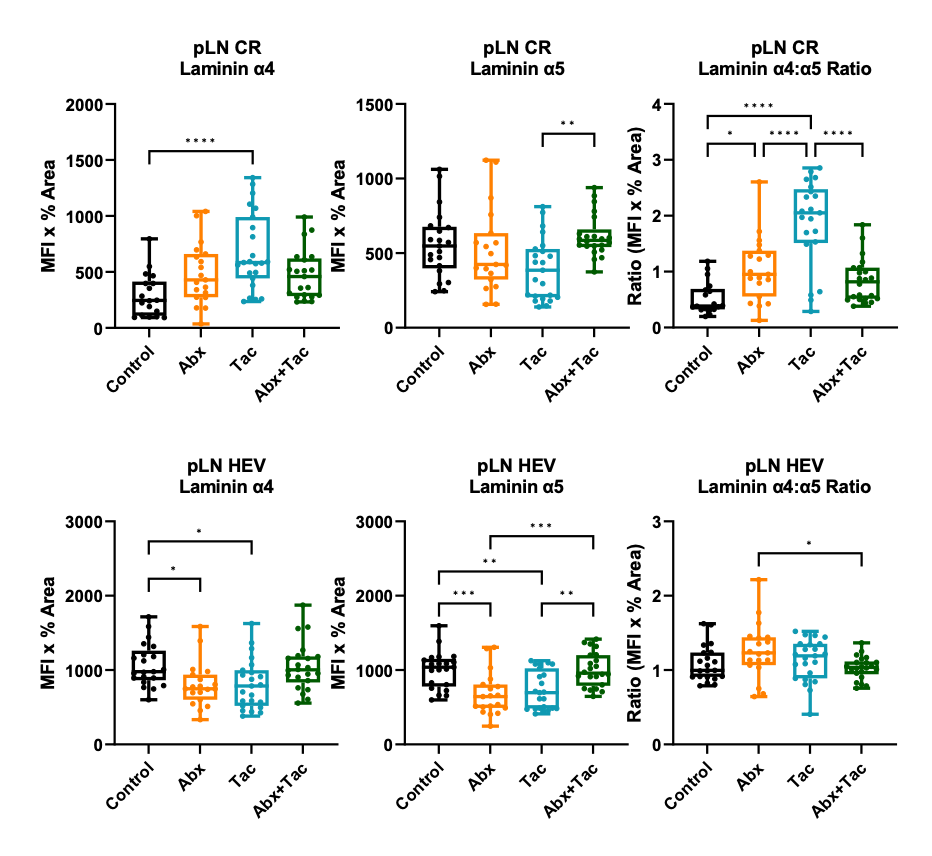
f. g. h.**

**S13.**


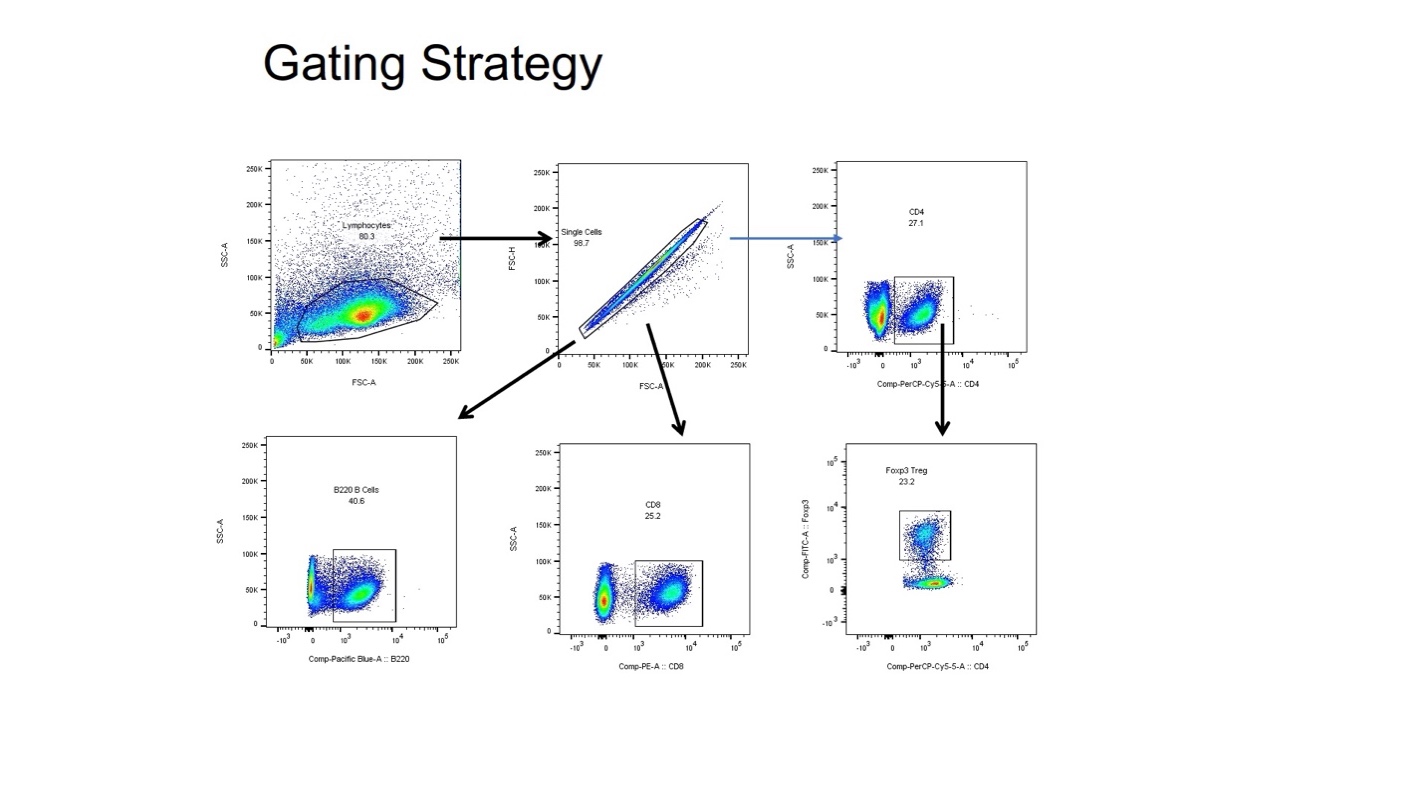


# Reference

Basu, S., Duren, W., Evans, C.R., Burant, C.F., Michailidis, G., and Karnovsky, A. (2017). Sparse network modeling and metscape-based visualization methods for the analysis of large-scale metabolomics data. Bioinformatics. 33(10), 1545-1553. DOI: 10.1093/bioinformatics/btx012.

Cao, Y., Dong, Q., Wang, D., Zhang, P., Liu, Y., and Niu, C. (2022). microbiomeMarker: an R/Bioconductor package for microbiome marker identification and visualization. Bioinformatics. 38(16), 4027-4029. DOI: 10.1093/bioinformatics/btac438.

Franzosa, E.A., McIver, L.J., Rahnavard, G., Thompson, L.R., Schirmer, M., Weingart, G., Lipson, K.S., Knight, R., Caporaso, J.G., Segata, N., et al. (2018). Species-level functional profiling of metagenomes and metatranscriptomes. Nat Methods. 15(11), 962-968. Published online 2018/11/01 DOI: 10.1038/s41592-018-0176-y.

Hattori, M., Tanaka, N., Kanehisa, M., and Goto, S. (2010). SIMCOMP/SUBCOMP: chemical structure search servers for network analyses. Nucleic Acids Res. 38(Web Server issue), W652-656. Published online 2010/05/13 DOI: 10.1093/nar/gkq367.

Kanehisa, M. (2017). Enzyme Annotation and Metabolic Reconstruction Using KEGG. Methods Mol Biol. 1611, 135-145. DOI: 10.1007/978-1-4939-7015-5_11.

Kanehisa, M., Goto, S., Sato, Y., Furumichi, M., and Tanabe, M. (2012). KEGG for integration and interpretation of large-scale molecular data sets. Nucleic Acids Res. 40(Database issue), D109-114. Published online 2011/11/15 DOI: 10.1093/nar/gkr988.

Kawamura, N., Shinoda, K., Sato, H., Sasaki, K., Suzuki, M., Yamaki, K., Fujimori, T., Yamamoto, H., Osei-Hyiaman, D., and Ohashi, Y. (2018). Plasma metabolome analysis of patients with major depressive disorder. Psychiatry Clin Neurosci. 72(5), 349-361. Published online 2018/01/23 DOI: 10.1111/pcn.12638.

Kieser, S., Zdobnov, E.M., and Trajkovski, M. (2021). Comprehensive mouse gut metagenome catalog reveals major difference to the human counterpart. bioRxiv. 2021.2003.2018.435958. DOI: 10.1101/2021.03.18.435958.

Kim, S., Chen, J., Cheng, T., Gindulyte, A., He, J., He, S., Li, Q., Shoemaker, B.A., Thiessen, P.A., Yu, B., et al. (2021). PubChem in 2021: new data content and improved web interfaces. Nucleic Acids Res. 49(D1), D1388-D1395. Published online 2020/11/06 DOI: 10.1093/nar/gkaa971.

Law, C.W., Chen, Y., Shi, W., and Smyth, G.K. (2014). voom: Precision weights unlock linear model analysis tools for RNA-seq read counts. Genome Biol. 15(2), R29. Published online 20140203 DOI: 10.1186/gb-2014-15-2-r29.

Matsumoto, M., Ebata, T., Hirooka, J., Hosoya, R., Inoue, N., Itami, S., Tsuji, K., Yaginuma, T., Muramatsu, K., Nakamura, A., et al. (2014). Antipruritic effects of the probiotic strain LKM512 in adults with atopic dermatitis. Ann Allergy Asthma Immunol. 113(2), 209-216 e207. Published online 2014/06/05 DOI: 10.1016/j.anai.2014.05.002.

McMurdie, P.J., and Holmes, S. (2013). phyloseq: an R package for reproducible interactive analysis and graphics of microbiome census data. PLoS One. 8(4), e61217. DOI: 10.1371/journal.pone.0061217.

Noecker, C., Eng, A., Srinivasan, S., Theriot, C.M., Young, V.B., Jansson, J.K., Fredricks, D.N., and Borenstein, E. (2016). Metabolic Model-Based Integration of Microbiome Taxonomic and Metabolomic Profiles Elucidates Mechanistic Links between Ecological and Metabolic Variation. mSystems. 1(1). Published online 2016/05/31 DOI: 10.1128/mSystems.00013-15.

Sugahara, H., Yao, R., Odamaki, T., and Xiao, J.Z. (2017). Differences between live and heat-killed bifidobacteria in the regulation of immune function and the intestinal environment. Benef Microbes. 8(3), 463-472. Published online 2017/04/27 DOI: 10.3920/BM2016.0158.

Taur, Y., 2023. yingtools2 package.

Wishart, D.S., Feunang, Y.D., Marcu, A., Guo, A.C., Liang, K., Vazquez-Fresno, R., Sajed, T., Johnson, D., Li, C., Karu, N., et al. (2018). HMDB 4.0: the human metabolome database for 2018. Nucleic Acids Res. 46(D1), D608-D617. Published online 2017/11/16 DOI: 10.1093/nar/gkx1089.

Xia, J., and Wishart, D.S. (2010). MSEA: a web-based tool to identify biologically meaningful patterns in quantitative metabolomic data. Nucleic Acids Res. 38(Web Server issue), W71-77. Published online 20100510 DOI: 10.1093/nar/gkq329.
